# Label-Free High-Resolution Photothermal Optical Infrared Spectroscopy for Spatiotemporal Chemical Analysis in Fresh, Hydrated Living Tissues and Embryos

**DOI:** 10.1021/jacs.3c08854

**Published:** 2023-11-02

**Authors:** Nika Gvazava, Sabine C. Konings, Efrain Cepeda-Prado, Valeriia Skoryk, Chimezie H. Umeano, Jiao Dong, Iran A. N. Silva, Daniella Rylander Ottosson, Nicholas D. Leigh, Darcy Elizabeth Wagner, Oxana Klementieva

**Affiliations:** † Department of Experimental Medical Science, Lund University, 22180 Lund, Sweden; ‡ MultiPark, Lund University, 22180 Lund, Sweden; § NanoLund, Lund University, 22180 Lund, Sweden; ∥ Department of Laboratory Medicine, Molecular Medicine and Gene Therapy, 22184 Lund, Sweden; ⊥ Lund Stem Cell Center, Lund University, 22100 Lund, Sweden; # Wallenberg Centre for Molecular Medicine, Lund University, 22184 Lund, Sweden

## Abstract

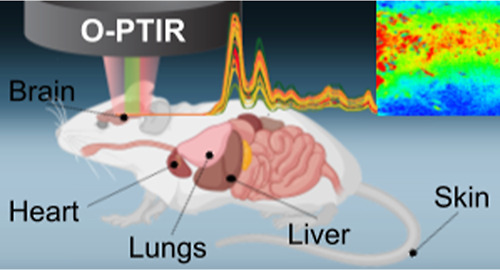

Label-free chemical imaging of living and functioning systems is the holy grail of biochemical research. However, existing techniques often require extensive sample preparation to remove interfering molecules such as water, rendering many molecular imaging techniques unsuitable for in situ structural studies. Here, we examined freshly extracted tissue biopsies and living small vertebrates at submicrometer resolution using optical photothermal infrared (O-PTIR) microspectroscopy and demonstrated the following major advances: (1) O-PTIR can be used for submicrometer structural analysis of unprocessed, fully hydrated tissue biopsies extracted from diverse organs, including living brain and lung tissues. (2) O-PTIR imaging can be performed on living organisms, such as salamander embryos, without compromising their further development. (3) Using O-PTIR, we tracked the structural changes of amyloids in functioning brain tissues over time, observing the appearance of newly formed amyloids for the first time. (4) Amyloid structures appeared altered following standard fixation and dehydration procedures. Thus, we demonstrate that O-PTIR enables time-resolved submicrometer in situ investigation of chemical and structural changes in diverse biomolecules in their native conditions, representing a technological breakthrough for in situ molecular imaging of biological samples.

## Introduction

Spatiotemporal changes in the chemical and structural composition of biomolecules underpin health and disease. However, submicrometer-level chemical and structural alterations often occur before disease onset and before morphological changes can be detected using standard tissue-level techniques (i.e., spatial proteomics, histology, or immunohistochemical staining). Therefore, alternative techniques such as infrared imaging have been increasingly used in biomedical research and clinical diagnostics. Infrared imaging can simultaneously provide information about diverse biological macromolecules (e.g., proteins, lipids, metabolites, DNA, and RNA) as well as insights into their structural conformations (e.g., protein secondary structure^1^). Specifically, infrared microspectroscopy (μIR) has been used to characterize the compositional and structural changes associated with the pathology of diverse diseases such as Alzheimer’s disease,^2^ cancer,^3^ septic arthritis,^4^ and many others.^5,6^ However, the application of μIR is significantly limited for biomedical studies since biological tissues and organisms contain high levels of water with strong IR absorption, which masks signals from protein vibrations. Thus, detecting biological molecules is known to be extremely limited in aqueous environments. In addition, the high degree of light scattering in the majority of biological tissues limits the use of thick tissues and necessitates that tissue is thinly sliced prior to analysis to visually correlate structures with μIR data. Therefore, biological tissues must typically be dehydrated and chemically fixed to be sectioned into thin slices (<10 μm) to be compatible with light-based microscopy visualization. This makes it impossible to study freshly extracted light-based biopsies, adding both time and further variables which may additionally complicate data analysis and interpretation. These limitations have thus far precluded the use of fresh, unprocessed biological tissues for μIR analysis. Overcoming these limitations would open the door to unprecedented, on-site rapid analysis suitable for clinical diagnostics as well as in vivo studies.

Optical photothermal infrared (O-PTIR) spectroscopy is an emerging IR-based technique that is revolutionizing molecular imaging by providing submicron spatial resolution independent of the diffraction limit of IR light.^7−14^ To overcome the diffraction limit of IR light, O-PTIR uses a visible probe beam to measure the photothermal responses caused by IR light, detecting intensity changes of the reflected (or transmitted) probe beam at a fixed wavelength of 532 nm.^15^ The fact that photothermal response is measured with a fixed wavelength beam provides a constant resolution which is 5–20 times better than traditional wavelength-dependent IR microspectroscopy techniques such as Fourier-transform (FT) IR and quantum cascade laser (QCL) IR.^7^ Importantly, when O-PTIR is used in reflectance mode, it becomes a surface-scanning technique, and therefore, sample thickness is irrelevant^16^ and, perhaps more importantly, substrate chemistry (critical for correlative multimodal imaging) becomes negligible.^16^ Moreover, O-PTIR has been successfully used to analyze hydrated living cells^17^ and nematodes,^7^ providing insights into lipid and protein distribution. While IR is considered nondestructive for biological samples, a certain concern exists due to the required use of high-intensity light beams (532 nm) that could impart thermal stress or light-induced injury, thus affecting tissue viability or function. In our proof-of-concept study, we asked whether the O-PTIR could be adapted for label-free rapid chemical and structural characterization of fresh, unprocessed tissue biopsies of diverse organs, as well as living and functional tissue slices, such as brain and living organisms.

Here, we demonstrate that the O-PTIR imaging parameters can be tuned to acquire interpretable IR spectra at imaging resolutions below the diffraction limit of IR light (i.e., submicrometer) in freshly prepared and hydrated biopsies, tissue slices, and living organisms without compromising sample viability and function. This includes real-time visualization of amyloid structure formation at submicrometer resolution in living, hydrated brain tissue slices derived from an animal model of Alzheimer’s disease, which has not been possible so far. Additionally, we show that tissue fixation and processing alter amyloid β-sheet structures. Therefore, O-PTIR holds potential as a rapid, label-free method with a high spatial resolution that can be used to evaluate molecular and structural changes in unprocessed and hydrated tissues and embryos for in vitro and in vivo diagnostics.

## Results

**Figure 1:**
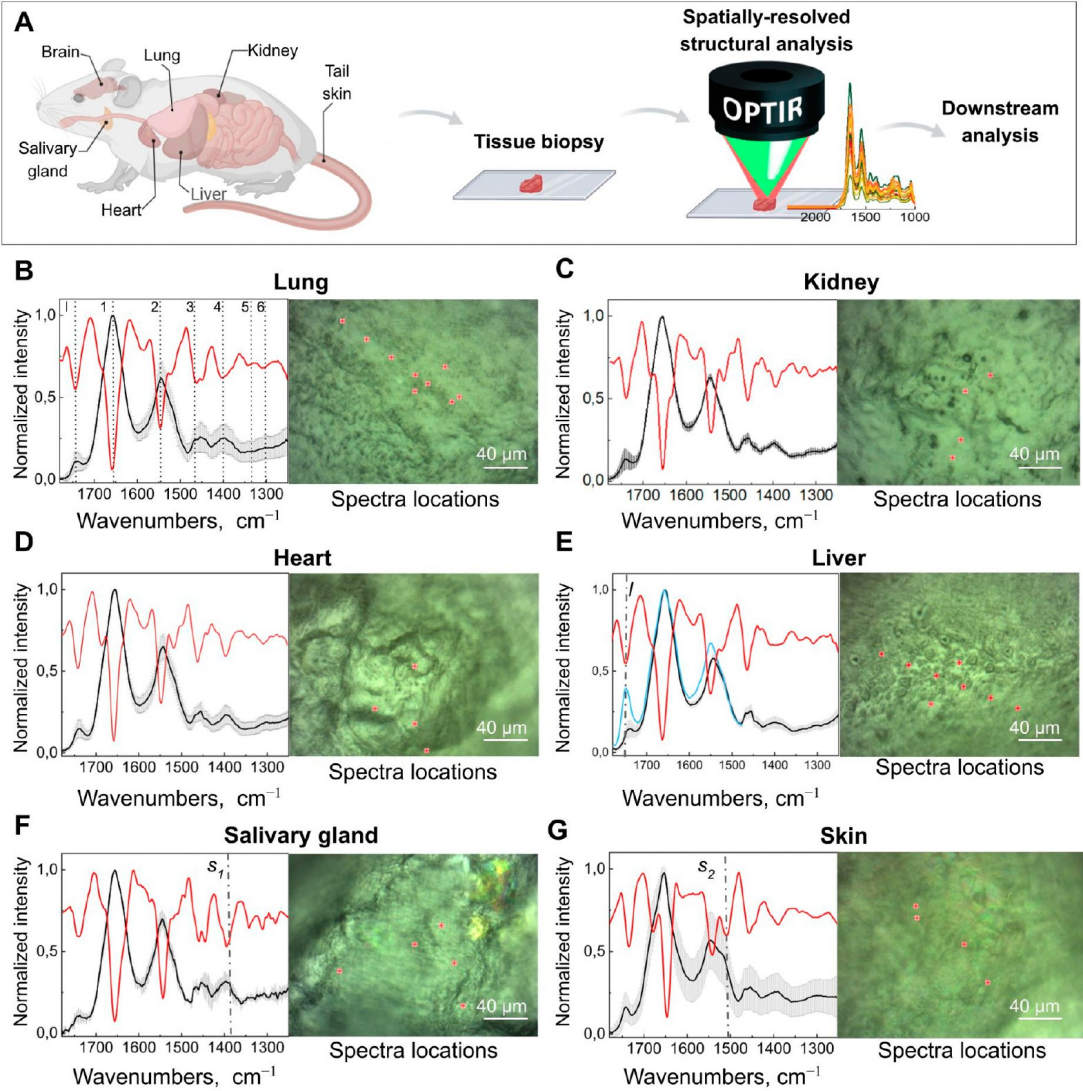
**O-PTIR of fresh tissue biopsies**. (**A**) Experimental flow (created with BioRender.com). **(**B–G**)** Sample analysis. Normalized and averaged O-PTIR spectra (black line) and second-derivative (red line) with representative bright-field images of the samples. The gray error bars represent s.d.; *N* = 3–10 areas from 3–4 tissue biopsies per organ. Red crosses indicate representative spectrum acquisition points, randomly selected for analysis. Peaks identified with second derivative spectroscopy are listed in Table 1. Bright-field images of tissue after spectroscopy are shown in Figure S1. In (C,F,G), dashed lines indicate bands that revealed a signal increase. In (E), the blue-colored spectrum trace was acquired at the location indicated in Supplemental Figure 2D,E and revealed a signal increase at 1740 cm^–1^, which corresponds to elevated ester levels.

To investigate the utility of O-PTIR in studying unprocessed tissue samples, we analyzed freshly extracted mouse tissue biopsies from several organs (lung, heart, kidney, liver, and salivary gland) and body parts (i.e., tail). The organ biopsies were placed in a 24-well plate with minimal nutritional support (i.e., DMEM/F12 media with 1% fetal bovine serum) and kept at room temperature for 10–30 min until measurements. For the O-PTIR measurements, tissue biopsies were placed directly on normal histological glass slides and examined without any tissue manipulations with an O-PTIR microscope (Figure 1A). We found that the combination of high-speed (1000 cm^–1^/s) laser scanning with attenuated IR power set to 22% can be used to acquire O-PTIR spectra with an IR-like quality (Figures 1B–G and S1). IR laser energies higher than 22% led to tissue damage and spectral artifacts, such as alterations of the amide II-to-amide I ratio (Figure S2). Thus, using 22% IR laser intensity, together with high-speed scan rates, it was possible to obtain spectra with low signal-to-noise ratio from all hydrated tissues. Using second derivatives, we identified expected typical peaks for proteins, lipids, and other metabolites (Figure 1B–G, Table 1). Further analyzing liver spectra, we noted distinct signal elevation at 1740 cm^–1^ in some regions of liver biopsies (Figure 1E, blue trace, dashed line l), which corresponds to ester groups (−C=O, −COOH) highly present in lipids; the observed intensity changes can indicate levels of lipidation^19^ or lipid oxidation^18^ and has been used for steatosis diagnosis using conventional FTIR-based spectroscopy.^19,20^ We also noted a significant increase in the peak area centered at 1452–1396 cm^–1^ (Figure 1F, dashed line S_1_) that has been described as methylene groups of proteins and lipids shown to be important for salivary gland characterization.^21^ We also recorded O-PTIR spectra directly from the mouse tail skin, allowing the detection of spectral bands, i.e., amide II (1500–1590 cm^–1^) and amide I (1600–1700 cm^–1^) and a prominent peak at 1515 cm^–1^ (Figure 1G, dashed line S_2_) that was previously described as a change in tyrosine vibrations.^22^ Thus, O-PTIR can be used to acquire spectroscopic information for label-free assessment of nonprocessed fresh and hydrated biopsies immediately after excision. Importantly, this includes the detection of bands, such as amides I and II, which overlap with water in traditional IR microscopic approaches and thus necessitate sample processing.

**Table 1:** Bands Identified from the Second Derivative of O-PTIR Spectra Corresponding to IR Bands Found in the Literature

	identified band (cm^–1^)	description in the literature^55^	examples of IR studies in relevant fields
l	1740	ester groups	liver steatosis^19,56^
1	1656	amide I (α-helix)	lung cancer^57^
			lung fibrosis^58^
2	1546	proteins (amide II band)	lung cancer^57^
S_2_	1515	tyrosine ring^1^	
3	1464	lipids, cholesterol esters, and cholesterol	lung cancer^57^
S_1_	1396	symmetric methyl (CH_3_) bending proteins, nucleic acids, and lipids	salivary gland^21^
4	1390		breast cancer^59^
5	1340	collagen	lung fibrosis^58^
6	1306	amide III/collagen	cardiac amyloidosis^60^
			breast cancer^61^

While IR spectroscopy itself has been considered to be “non-destructive” and can be used to image biological samples over time ranging from a few milliseconds to hours,^23^ O-PTIR uses two colinear infrared and visible lasers. Irradiation using one or both of these lasers could potentially damage and/or affect sample viability.^24^ While O-PTIR has been successfully used for microscopy-based spectroscopy of cells^17^ in vitro and *Caenorhabditis elegans* as experimental end points,^7^ it has not yet been reported if O-PTIR damages living tissue or organisms. Therefore, we examined metabolic activity after the collection of the O-PTIR spectra using a water-soluble tetrazolium-1 (WST1) assay and found that tissues retained metabolic activity after O-PTIR irradiation at levels similar to controls, indicating maintenance of tissue viability (Figure S3). As a next step for biopsy characterization, we performed standard histological analysis (i.e., from formalin-fixed and paraffin-embedded samples) of O-PTIR exposed tissues and compared these to biopsies taken from the same tissue just before O-PTIR exposure. We did not observe any major tissue damage or signs of cellular necrosis, indicating that our settings for the O-PTIR measurements did not grossly damage the tissue biopsy and, importantly, that routine clinical assessment using histology can be employed as a downstream analysis after the O-PTIR measurements (Figure S4).

**Figure 2:**
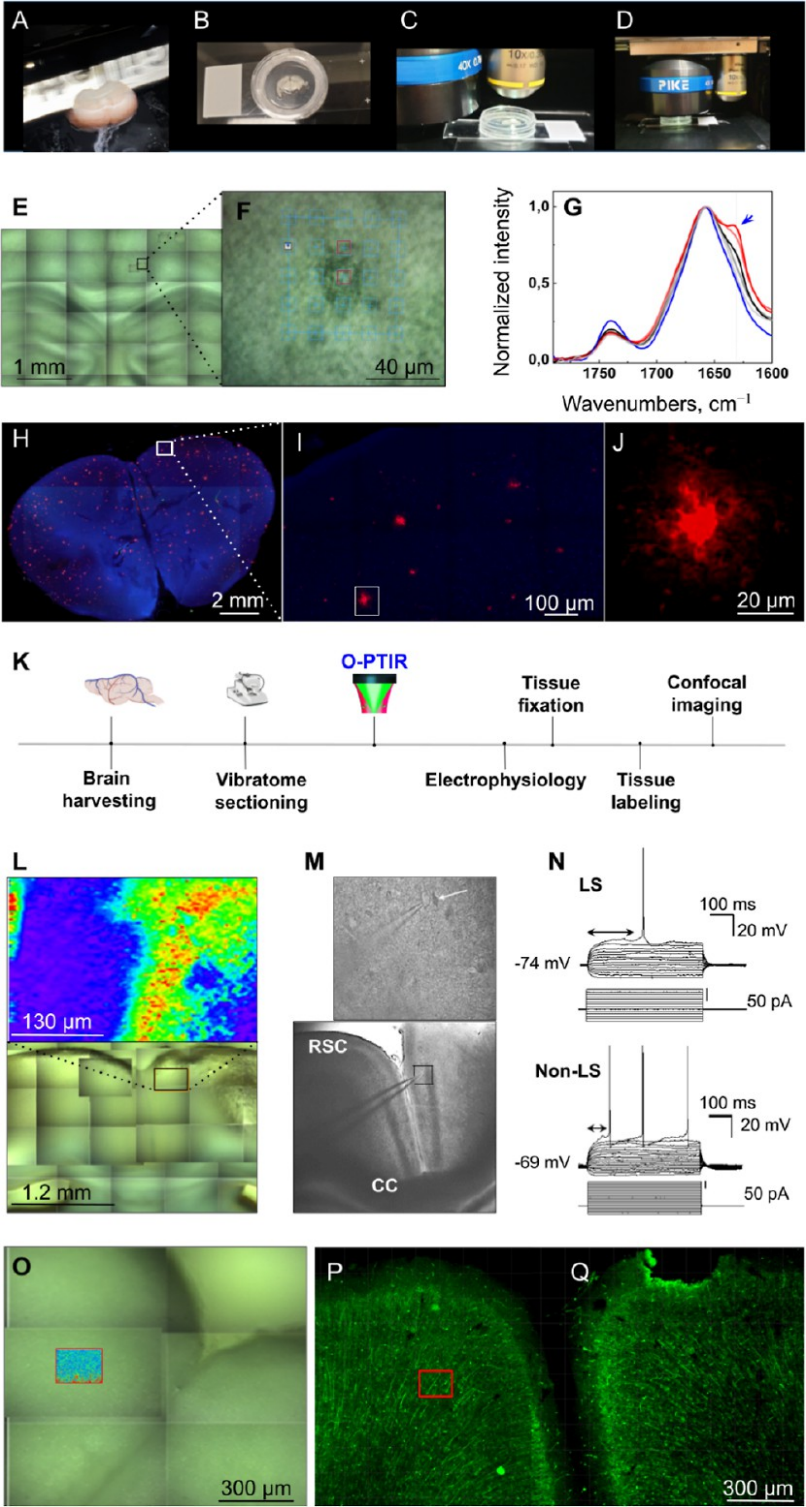
**Imaging of amyloid structures in living brain tissue at submicron resolution**. (**A**) Vibratome-assisted slicing (275 μm thickness) of fresh murine brain tissue. (**B**) Incubation of tissue sections in a custom imaging setup with oxygenated artificial CSF (‘brain media’) at room temperature. (**C,D**) the custom-made chamber under O-PTIR low (15×) and high (60×) objectives. (**E**) Hydrated brain tissue was imaged after O-PTIR measurements using a Cytation5 multimode imager. Black square indicates the O-PTIR measurement area. (**F**) Higher magnification image of the area in (E), with spectral positions. (**G**) Normalized O-PTIR spectra collected from the positions indicated in (F). Arrow indicates 1630 cm^–1^ shoulder characteristic to amyloid fibrils.^1^ Red spectra correspond to the positions indicated by red squares. (**H**) Amyloid plaques were stained with Amytracker520^R^ after O-PTIR measurements, and the DAPI filter settings were used to collect tissue autofluorescence after the tissue was fixed with 4% PFA. The brain tissue slice was imaged using a Cytation5 multimode imager. Red dots are amyloid plaques. (**I**) Higher magnification image of the same area in (E) and acquired with confocal microscopy, with amyloid plaques stained in red. (**J**) High-resolution confocal image of amyloid plaque selected in E and imaged with confocal microscopy. (**K**) Experimental flow (created with BioRender.com). (**L**) Lower panel: Bright-field image showing the location (upper panel) of a single energy map acquired at 1656 cm^–1^ in the retrosplenial cortex (RSC). (**M**) Differential interface contrast (DIC) images showing the position of the electrode during the electrophysiological recording in RSC of acute (living) tissue slices exposed to O-PTIR. Lower panel is an overview; the upper panel shows the neurons located in the tissue. (**N**) Voltage responses to current injection of two different pyramidal neurons recorded in current clamp mode after O-PTIR measurements. Late-spiking (LS) and non-late spiking pyramidal cell (non-LS). (**O**) Bright-field image showing the location of a single energy map acquired at 1656 cm^–1^ in RSC (the insert). (**P,Q**) Confocal images of RSC after O-PTIR in one hemisphere and unexposed hemisphere. Red rectangle indicates the location of O-PTIR measurements. The neurons were labeled with the neuronal marker MAP2 (green). High-magnification images are shown in Figure S8.

Highly encouraged by our findings of the preserved viability of tissues, we designed and optimized our setup to allow for the O-PTIR imaging of living brain tissue slices of defined thickness (Figure 2A–D), including tissue functional analysis and electrophysiology, after the acquisition of the O-PTIR spectra. We made a custom sample support chamber for brain tissue slices (Figure S5) to allow ex vivo culture in a *N*-methyl-d-glucamine (NMDG) artificial cerebrospinal fluid (aCSF) media formulation, which has been shown to preserve neuronal function over short time frames.^25^ Since metabolic activity alone is insufficient for assessing cellular-level functions, acute brain slices were prepared for electrophysiological recordings using whole-cell patch-clamp to evaluate neuronal activity in brain tissues after O-PTIR measurements. We observed a robust firing pattern of action potentials as well as activation of voltage-gated sodium and potassium channels in cortical neurons, similar to patterns reported in the literature for normal brain slices at the location of the O-PTIR imaging-based measurements (Figure S6).

Once the parameters for the O-PTIR measurements in living tissue were established, acute brain slices from the APP/PS1 Alzheimer mouse model were used to detect structural changes in amyloid-β proteins. Due to the transgenic expression of mouse/human amyloid precursor protein (APP) and a mutant human presenilin 1 (PS1) in the brain, APP/PS1 mice overexpress aggregation-prone amyloid-β proteins that spontaneously form amyloid plaques in brain parenchyma.^26^ We used freshly extracted APP/PS1 mouse brains from animals of 12 months that were cut into 275 μm thick brain slices. To keep brain tissue alive, slices were kept in an oxygenated buffer at room temperature (Figure 2A–D). We examined cortex tissue, which consistently has β-sheet-rich amyloid plaques at that age. Plaques were located using fast hyperspectral array scanning over an extended area (10 spectra with 50 μm step, 1 s per spectrum, over 1600–1700 cm^–1^). We identified plaques by a shoulder between 1640 and 1620 cm^–1^ that has been previously identified to correspond to β-sheet structrures^27^ (Figure 2E–G, S7). Following O-PTIR spectroscopy, we stained APP/PS1 brain slices using a dye, Amytracker^R^ (EbbaBiotech, Solna, Sweden), a luminescent conjugated polyelectrolyte probe specific to amyloids,^28^ and confirmed the presence of amyloid plaques using conventional and confocal fluorescence microscopy in the same slices used for O-PTIR imaging (Figure 2H–J). Thus, we provide the first evidence that the O-PTIR imaging can successfully detect amyloid plaques in freshly prepared, hydrated brain tissue slices.

To perform the measurements in living tissue, we first sought to better characterize the extent of the local damage induced by the O-PTIR imaging. Due to the difficulty of reidentifying the exact region from which spectra were collected, we chose to target the retrosplenial cortex (RSC), a small dorsomedial parietal area in the cortex, as it can easily be identified under both microscopes and is characterized by two distinct populations of pyramidal cells with different firing patterns, late spiking (LS) (long time to first spike) and nonlate (non-LS) spiking cells, due to the ease of reimaging/identifying this region. First, we produced single-energy maps at 1656 cm^–1^ (i.e., maximum O-PTIR intensity in brain spectra) in the RSC using our custom-made sample support (Figure S5) to permit brain slices of defined thickness to be imaged under aqueous conditions. Submerged tissue slices were placed into the sample support and covered by an IR-transparent 0.1 mm CaF_2_ window, which allowed the objective to reach the tissue while maintaining a minimal amount of buffer around the brain slice. O-PTIR single-energy maps of 250 × 400 μm were acquired using the same O-PTIR settings with a step size of 500 nm, the maximum resolution of the instrument. Bright-field images of the RSC both in the electrophysiology setup and in the O-PTIR microscope confirm the ability to readily detect distinct regions within the RSC (Figures 2L–M and S6), with which we acquired single-energy maps at 1656 cm^–1^ (Figure 2L). Firing patterns of pyramidal cells in the RSC were identified after O-PTIR (Figure 2N), confirming the retention of functional cells in the irradiated area. To further investigate the extent of damage using specific molecular markers which can precede loss of cellular or tissue function, tissue slices were fixed, processed^29^ after O-PTIR imaging, and labeled with microtubule-associated protein 2 (Map2), a dendritic marker,^30^ to evaluate the morphological condition of neurons after O-PTIR imaging (Figures 2O–Q and S8A–E). We also stained the tissue with AnnexinV, which binds to phosphatidylserine residues that are translocated in the membrane and are externalized during apoptosis (Figure S8F–I). We did not observe any major loss of neurons or alteration of Map2 integrity staining after the O-PTIR imaging. Furthermore, there was no noticeable increase in the level of Annexin V in these same regions. Thus, the O-PTIR does not seem to induce major tissue damage immediately after irradiation in the regions in which imaging occurs.

**Figure 3:**
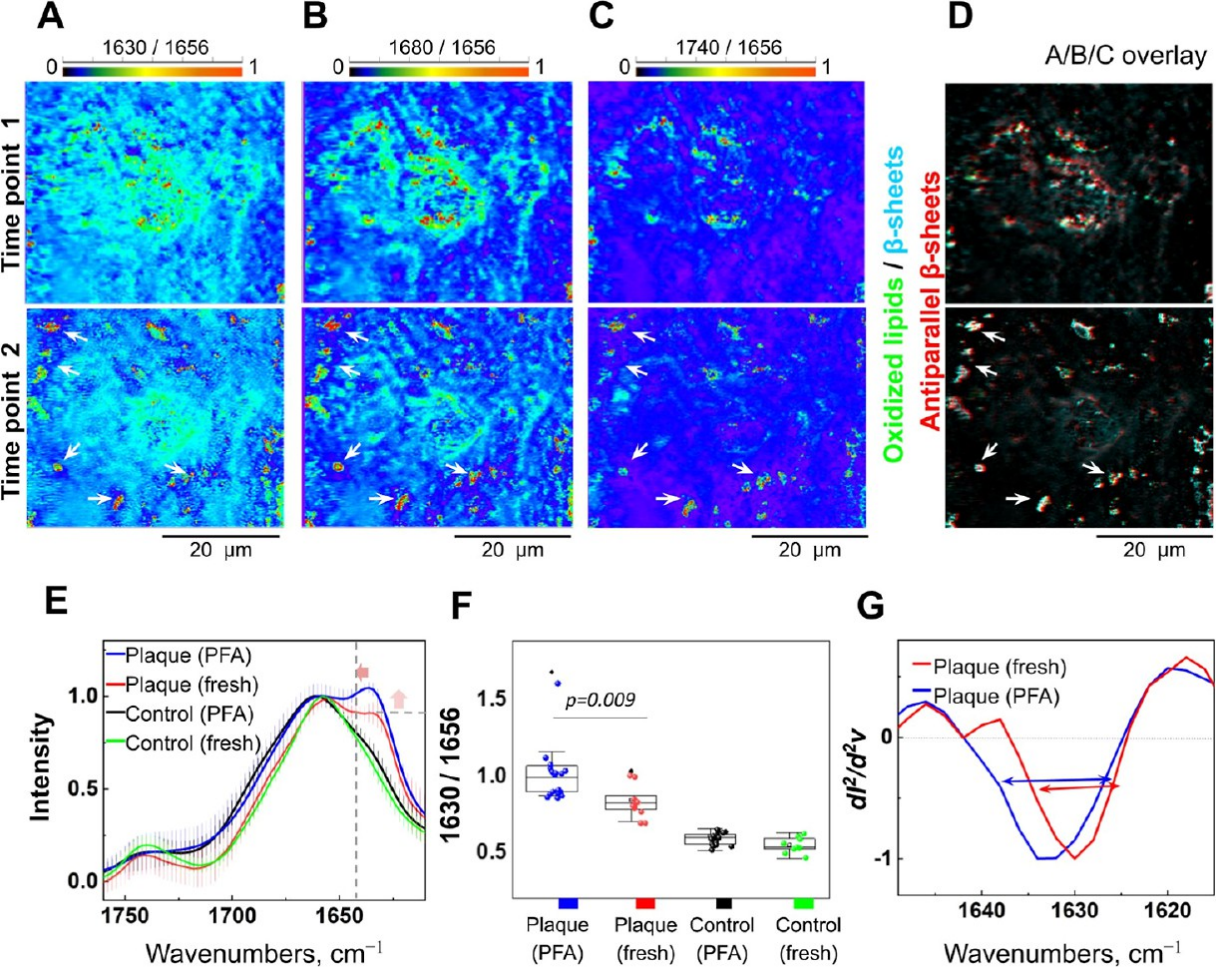
**Time-resolved imaging of amyloid structures in living tissue at submicron resolution**. (**A-C**) Single-frequency O-PTIR maps of the same spot in living brain tissue at time 0 and time 10 min. (**A**) Change of β-sheet structure distribution over time, calculated as a ratio of 1630 cm^–1^ (the main β-sheet band) to 1656 cm^–1^ signal (Amide I maximum). Lower panel, white arrows indicate newly formed β-sheet structures. (**B**) Change of distribution of fibrillar β-sheet structures over time, calculated as a ratio of 1680 cm^–1^ (antiparallel β-sheets) to 1656 cm^–1^ intensity. Lower panel, white arrows indicate newly formed antiparallel β-sheets. (**C**) Change in lipid oxidation over time, calculated as a ratio of 1740 cm^–1^ (R-CO-OR groups) to 1656 cm^–1^ intensity. Lower panel, white arrows indicate new spots of lipid oxidation. (D) Overlay of (A-C) single-frequency IR maps, white arrows indicate colocalization of newly formed antiparallel β-sheets and oxidized lipids. The scale bar is the same for (A-D). (**E**) Normalized and averaged O-PTIR spectra of fresh and tissue fixed in 4% PFA. Arrows indicate an increase in the intensity of β-sheet (up) and changes in structural content; band position shift from 1630 cm^–1^ to 1634 cm^–1^. Error bars are s.d. (F) β-sheet signal intensity in living and fixed tissue. Tukey’s post-hoc test; *N* = 8–10 spots. Data are the mean ± s.d. (G) Second derivatives of IR absorbance spectra. Arrows indicate changes in bandwidth.

Next, we took advantage of the superior submicrometer resolution capability of the O-PTIR (Figure S9) and our optimized chamber, allowing us to keep the tissue hydrated during the O-PTIR for measurement and monitoring structural features in living brain tissue over time. Thus, we tested O-PTIR utility for time-resolved observation of the same amyloid plaque in brain slices from APP/PS1 mice. We acquired and compared multiple single-frequency IR maps from the same area to record the distribution of specific chemical features in the tissue over time. To visualize β-sheet structures, we acquired IR maps at the frequencies of 1656, 1630, and 1680 cm^–1^, a double-band feature for amyloid proteins, which is consistent with the accepted IR signature for antiparallel β-sheets.^31−33^ Since oxidized lipids have been previously associated with amyloid structures,^13,18,34^ we also acquired a map at the frequency of 1740 cm^–1^ to detect oxidized lipids using the 1656 cm^–1^ map as a denominator for corresponding ratio maps (Figure 3A–C). Thus, we obtained and compared four different spectral maps showing the distribution of chemical features at two distinct time points (2.5 min per map; 10 min between each spectral map collected at each time point). Strikingly, we observed the formation of new small spots with β-sheet structures observed in the time point (2 images indicated by arrows in the lower panel of Figure 3A) with a high content of antiparallel β-sheet structures^35,36^ (Figure 3B lower panel, arrows). The appearance of new β-sheet structures was accompanied by an increase in the presence of oxidized lipids that accompany amyloid aggregation in AD tissue^18^ (Figure 3C,D). After O-PTIR, brain tissue that was irradiated by O-PTIR was fixed and labeled with the antibody sensitive to amyloid-β proteins, 82E1. Using confocal microscopy, we observed an increased amount of small immunolabeled clusters as compared with the control tissue, an adjacent tissue slice that was not exposed to the O-PTIR (Figure S10).

We next compared the O-PTIR-acquired spectra of amyloid plaques in living brain tissue with adjacent tissue slices from the same mouse that were chemically fixed with 4% (v/v) paraformaldehyde (PFA). We observed a lower intensity of the β-sheet signal in fresh tissue as compared to the PFA-fixed tissue and a shift of the signal maximum from 1630 cm^–1^ in fresh tissue to 1634 cm^–1^ in PFA-fixed tissue (Figure 3E,F). In addition, we observed changes at the 1630 cm^–1^ bandwidth (Figure 3G), as detected by analyzing the second derivative of IR spectra.^37^ These striking observations of changes in β-sheet organization^36^ indicate that amyloid structures in living tissue can structurally differ from chemically processed ones. This points to the need to perform structural studies directly in living tissue, e.g., to assess antiamyloid therapies in living brain tissue using recently tested lecanemab^38^ or aducanumab.^39^

**Figure 4:**
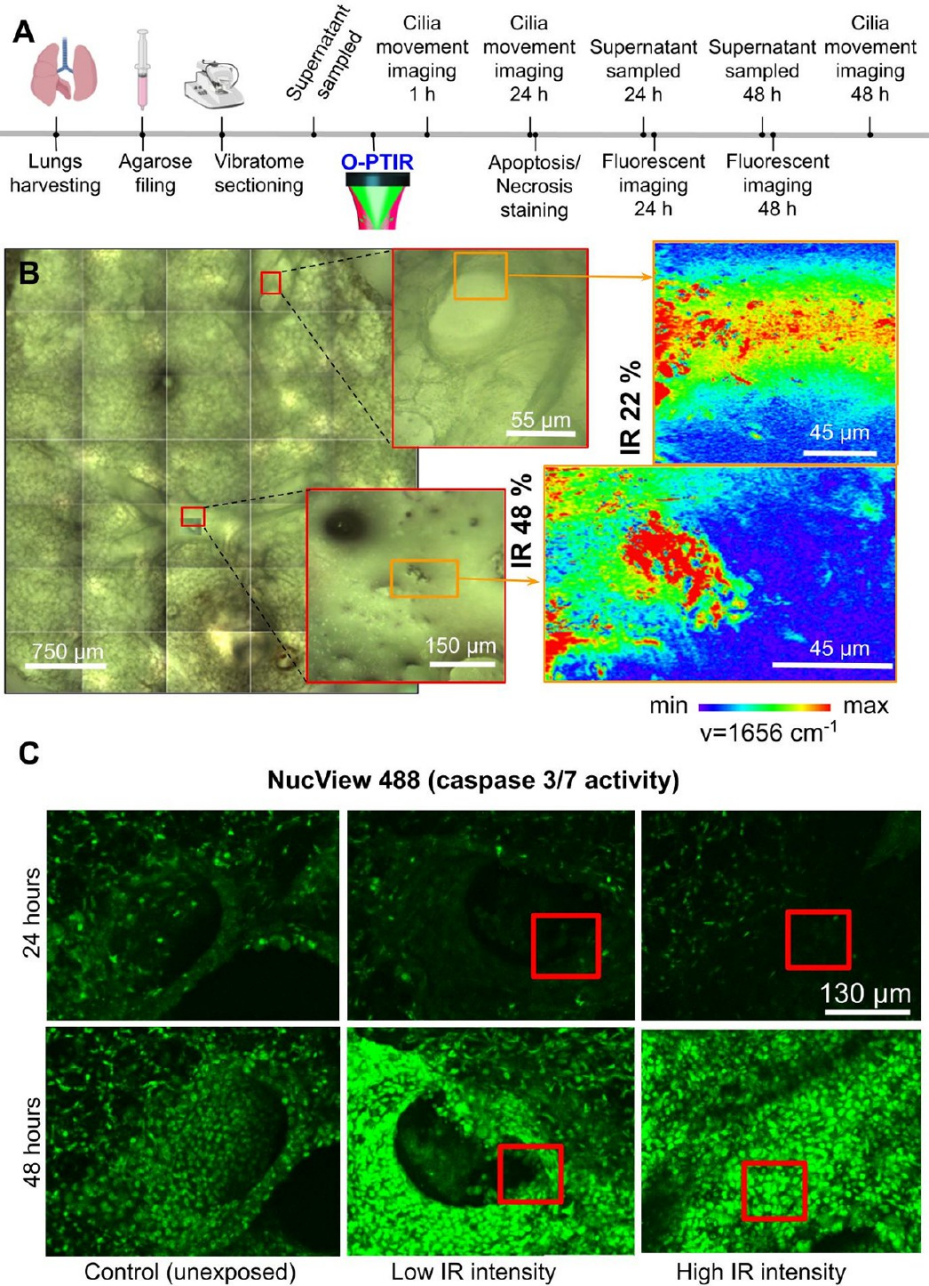
**Live tracking of cellular damage in precision-cut lung slices (PCLS) following O-PTIR imaging**. (**A**) Experimental overview (created with BioRender.com). Supernatant sampled for LDH assay as shown in Figure S11A. (**B**) Bright-field overview of 4 mm PCLS punch with insets indicating airways subjected to hyperspectral imaging at 1656 cm^–1^ with different laser intensities. (**C**) Maximum projection intensity from z-stacks acquired with confocal microscopy and stained live with NucView 488 (fluorescent DNA dye coupled to a peptide sequence recognized by active caspase 3/7). Red insets represent the areas imaged with O-PTIR at different laser intensities and tracked over time for 24 and 48 h, showing subjective increases in staining at 48 h in samples subjected to O-PTIR imaging. Control samples represent similarly cultured and transported samples that were not exposed to O-PTIR.

While we did not observe evidence of apoptosis (i.e., AnnexinV staining, Figure S8) or loss of neuronal functioning after the O-PTIR in brain tissue slices after short time frames (i.e., hours), irradiation-induced effects may take longer to be detectable in tissues as this is a complex cellular response. Furthermore, it is extremely challenging to track or identify the exact positions of spectroscopic measurements in optically translucent tissues, such as brain tissue. Therefore, to better understand the extent of local damage induced by O-PTIR irradiation, we utilized lung tissue inflated with agarose to generate precision-cut lung slices (PCLS). PCLS offer the distinct benefit that they become semitransparent in the airspaces with the addition of agarose and therefore retain identifiable morphological features in bright-field microscopy. Furthermore, protocols have been developed which allow for extended and functional ex vivo culture over several days (e.g., retention of ciliary beating in airways for up to 1 week).^40^ PCLS thus permit longer time windows with which to assess cellular damage. After generating agarose-filled murine PCLS, we identified morphologically distinct airways in each slice and exposed these airways to the O-PTIR imaging at 22 or 48% IR power (Figure 4A,B). We monitored these same airways for 48 h using live imaging of ciliary beating in the airways as well as noncytotoxic, live cell fluorescent tracers of cellular damage to allow for the tracking of these same regions over time: NucView488, a fluorogenic DNA dye coupled to a caspase 3/7 recognition sequence (indicative of early apoptosis), and RedDot2, a far-red nuclear dye staining membrane-compromised cells. While we noted subjective increases in the general amount of cells expressing active cleaved-caspase 3/7 activity in airways exposed to O-PTIR and significant increases in lactase dehydrogenase release at 48 h (Figures 4C and S11), we did not observe any notable impairment in ciliary beating in these same irradiated airways over 48 h (Videos S1 and S2). This indicates that while O-PTIR imaging may induce some degree of local cellular damage, this damage does not cause widespread functional impairment in these specific regions.

**Figure 5:**
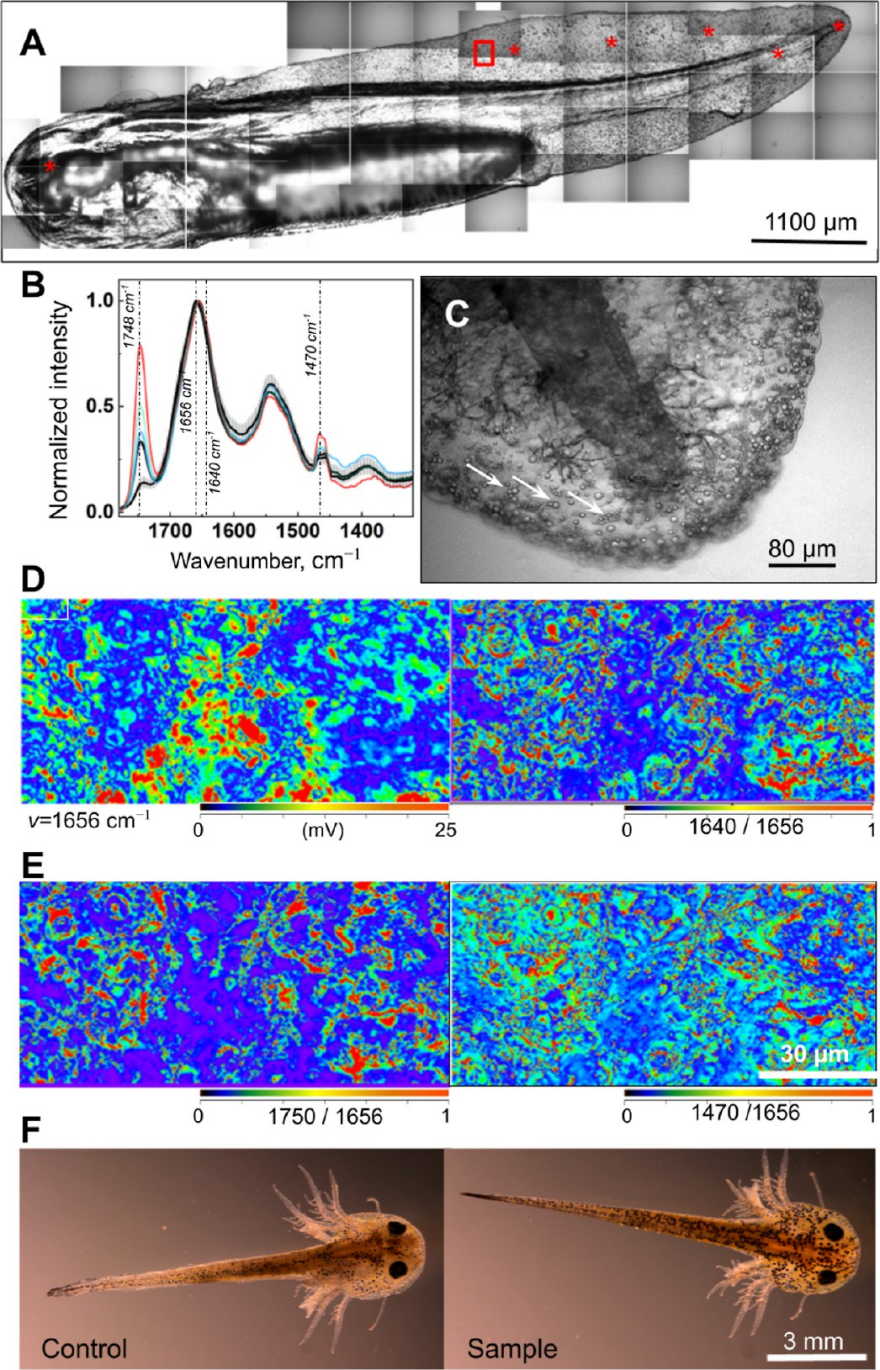
***In vivo* O-PTIR imaging of lipids and proteins in *P. waltl.***(**A**) Bright-field mosaic of the *P. waltl* embryo. Asterisks indicate the O-PTIR spectrum acquisition points. Red rectangle indicates the O-PTIR map position shown in (D) and (E). (**B**) Normalized O-PTIR spectra acquired at different locations are shown in (A). Black traces, spectra acquired from spots outside lipid droplets; error bars, s.d. for black-trace spectra; *N* = 6 animals. Colored traces are spectra acquired from the lipid droplets. Dashed lines show the wavenumber for single-frequency IR maps in (D) and (E). (**C**) Bright-field image of the embryonic tail. Arrows indicate lipid droplets on the skin. Spectra for the droplet and area outside the droplet are shown in Figure S12C. (**D**) Single-frequency IR maps were acquired at the same spot at two energy levels corresponding to proteins (1656 cm^–1^ and 1640 cm^–1^). (**E**) Single-frequency IR maps acquired at the same spot at two energy levels corresponding to R-CO-OR groups (1750 cm^–1^ and 1470 cm^–1^), showing the distribution of lipids. (**F**) Bright-field images of *P. waltl* embryos 12 d after O-PTIR measurements (‘Sample’). Compared to the control animals not analyzed by O-PTIR, no change in animal development was apparent.

To further explore the potential of the O-PTIR as a nondestructive technique for in vivo imaging, we analyzed embryos of vertebrate *Pleurodeles waltl*, a commonly used species in developmental and regenerative biology (Figure 5A). *P. waltl* undergo embryonic development for ∼2 weeks, followed by ∼10 weeks as larvae before undergoing metamorphosis and living up to 20 years. During development, they proceed through well-characterized stages.^41^ Stage 34 was sampled in this study, which occurs at ∼11–13 days post fertilization (dpf). At this point, these animals have internal organs and their limbs are beginning to develop. We chose this stage as embryonic development provides a highly sensitive time window and any widespread tissue damage would likely manifest in altered development. First, we acquired O-PTIR spectra from different regions of the developing embryo (Figure 5A, red insets) and identified the main signal peaks to be at 1748, 1656, 1640, and 1470 cm^–1^ (Figure 5B).^42^ During the measurements, we microscopically observed droplets on the larva skin that were reminiscent of oil droplets. In order to verify their chemical content, we took advantage of the high spatial resolution of O-PTIR and acquired pinpoint spectra from these droplets (Figures 5C and S12). Chemical analysis revealed a strong peak centered at 1748 cm^–1^, indicating a high amount of esters (−C=O, −C=OOH), characteristic of lipids.^43^ Interestingly, we also observed a significant peak shift from 1740 to 1748 cm^–1^, potentially indicating enrichment in −C=OOH or, alternatively, the presence of a specific interaction that can shift the ester band 10 cm^–1^ up (Figure 5B,C). Further complementary analytical techniques and experiments are needed to provide more comprehensive information about the content of these droplets as well as their origin. Next, we tuned the wavelength to 1748, 1656, 1640, and 1470 cm^–1^ and constructed chemical maps of the dorsal side of the embryo (Figure 5D,E). Both lipid droplets and proteins were visible on single-frequency IR maps at 1750 and 1656 cm^–1^, respectively (Figure 5E). We reasoned that sampling along the tail would potentially lead to defects in tail development. However, we did not identify any gross defects in morphology or tail length, as assessed by the full length of the embryo. Importantly, all embryos survived the O-PTIR (*n* = 6) and developed similarly to controls not exposed to the O-PTIR (*n* = 6) (Figure 5F). Therefore, intensities used with O-PTIR do not cause tissue- or organism-level damage which prevents normal embryonic development and maturation. This indicates that the label-free nature of the O-PTIR allows for in vivo spectroscopic investigation of biological molecules that would otherwise require sacrificing animal and chemical tissue processing, altering structure and composition.

## Discussion

Specifically, we demonstrate that O-PTIR can be used as a novel IR imaging platform for the acquisition of time-resolved imaging of lipids and proteins in fresh biopsies of diverse tissues as well as in living brain tissue and whole organisms with submicron spatial resolution.^12^ Importantly, the O-PTIR resolution is 10 times better than that of μIR, i.e., approximately 400 nm for the O-PTIR vs 4.6 μm for the conventional μIR, at 1630 cm^–1^ corresponding to β-sheet structures and using the Rayleigh criterion of 0.61λ/NA (where λ is a wavelength and NA is the numerical aperture of the objective, here equal to 0.8). This opens up the possibility of visualizing biomolecular structures at submicron resolution in living tissue and, for example, of monitoring the onset, structural evolution, and spread of amyloid β-sheet aggregates in living brain tissue.

Our preliminary O-PTIR experiments have revealed the alteration of amyloid β-sheet structures in living brain tissue slices derived from APP/PS1 mice, a well-characterized animal model of Alzheimer’s disease. This murine model of AD is commonly used to study extracellular amyloid depositions in brain tissue known as amyloid plaques. We observed the formation of new antiparallel β-sheets, accompanied by changes in the intensity of stretching vibrations of ester bonds between phospholipids, which is important for synaptic neurotransmission and plasticity.^44^ As the observed intensity changes indicate the rapid formation of β-sheet structures and oxidized lipids, the combination of 1740, 1680, 1656, and 1630 cm^–1^ can be used as a biomarker to monitor local damage in living tissues at subcellular resolution. To the best of our knowledge, this is the first real-time visualization of amyloids in the act of their formation in living tissue, covisualized with damaging surrounding lipids. Although additional studies are needed to understand this phenomenon, we hypothesize that oxygen or temperature-induced changes due to laser exposure directly or indirectly triggered the formation of new amyloid β-sheet aggregates (Figure 3). Recent work has shown that small changes in temperature (∼2 °C) due to, for example, exothermic Aβ42 elongation, are capable of accelerating the nucleation process of new amyloids.^45^ Alternatively, oxidation has been shown to be associated with amyloid formation. Future work is necessary to elucidate the exact mechanism of amyloid formation in this setup and could include the use of antioxidants or free radical scavengers in the culture media to assess the role of oxidation. Assessment of local temperature changes is also critical for better understanding the actual temperature increases that occur during the O-PTIR imaging.

Our O-PTIR results support previous works showing that amyloid plaques can be detected in fresh samples of APP/PS1 mouse brain tissue using stimulated Raman scattering microscopy.^46,47^ Recent advances in instrumentation that combine O-PTIR and Raman spectroscopy in one instrument will be invaluable for better understanding the differential capabilities of both imaging modalities on the same tissue sample and thus remove the potential differences arising due to differences in sample preparation between studies. Furthermore, we demonstrate a new possibility for time-resolved images of living tissues.

As O-PTIR allows for the collection of IR spectra in fresh tissue samples and living organisms, it is also a promising tool for use as an in vivo multiomics tool for obtaining comprehensive information on various subcellular components (proteins, lipids, metabolites, DNA, RNA, etc.). Furthermore, as biological molecules are highly conserved across evolution, O-PTIR opens up new opportunities for biological tissues and organisms that currently have limited research tools (e.g., model organisms such as *P. waltl* or research species with limited species-specific antibodies). Lipid droplets on *P. waltl* embryonic skin have not been reported to date, but typically, lipids play a role in regulating transepidermal water loss.^48^ Alternatively, we hypothesize that they may act as nutrient stores during embryonic development in addition to the yolk sac or serve to secrete metabolites in a sequestered format to prevent subsequent uptake by the developing organism. Further studies are needed to define the specific compositions of these droplets and elucidate their biological importance. In general, we show that O-PTIR is well-tolerated by developing *P. waltl* embryos and that it is a promising new technique to study live animals in a label-free manner without the need for extensive sample processing.

In the future, an exciting potential area of application for O-PTIR imaging will be rapid molecule-based diagnosis of clinical diseases, similar to current approaches using IR-based spectroscopy for disease diagnosis.^49^ On-site O-PTIR analysis removes the need for sample preparation and could be used to rapidly screen biopsies during cancer surgery, such as Mohs surgery for skin cancer,^50^ substantially cutting down on the analysis and surgical time. Other potential clinical applications will rely on further instrument development. There is a substantial clinical need for techniques that can be used to evaluate intact organs without taking a biopsy (e.g., evaluation of donor organs prior to transplantation). The application of O-PTIR to whole, intact organs or larger organisms would require further technical developments, such as an adjustable sample stage or flexible or portable optics for the analysis of samples larger than a few centimeters thick.

Notwithstanding our promising findings, O-PTIR can be time-consuming as a standalone technique, which may make it suboptimal for assessing some living samples. Further instrument and accessory developments are needed to overcome this limitation. As one example, instruments combining the O-PTIR and epifluorescence modules could be used to decrease the imaging acquisition time, as specific antibodies for immunolabeling or transgenic animals containing fluorescent proteins could be used to rapidly locate immunolabeled areas. In addition to a reduction in acquisition time, such a development could also add specificity for spectroscopic measurements.

## Conclusions

We demonstrate that O-PTIR using modified imaging acquisition parameters allows it to be used as a novel IR imaging platform to provide submicron spatial resolution, enabling imaging of biological molecules such as lipids and proteins in hydrated, living tissues and organisms, as well as insights into their structural conformations. As a proof-of-concept, we conducted multiple experiments on freshly extracted tissue biopsies and living lung and brain tissue. Experiments on living lungs and brain tissues demonstrated unprecedented possibilities for obtaining structural information. By monitoring the formation of new antiparallel β-sheets, accompanied by changes in the intensity of stretching vibrations of ester bonds between phospholipids, we demonstrated the utility of O-PTIR as a novel multiomic approach to recording spectra directly from fresh, unprocessed biological samples.

## Methods

### Preparation of Mouse Brain and Lung Slices

All mouse experiments were compliant with the requirements of the Ethical Committee of Lund University (M11908–19). APP/PS1 mice (hAPPswe, PSEN 1dE985Dbo/Mmjax) were obtained from Jackson Laboratories, USA, and all mice were screened for the presence of the human APP695 transgene by PCR.

Briefly, the mice were anesthetized intraperitoneally with sodium pentobarbital (40–50 mg/kg) and transcardially perfused with ∼20 mL of oxygenated (carbogen, 5% CO_2_, 95% O_2_) *N*-methyl-d-glucamine (NMDG)-HEPES artificial cerebrospinal fluid (CSF) containing 92 mM NMDG, 2.5 mM KCl, 1.25 mM NaH_2_PO_4_, 30 mM NaHCO_3_, 20 mM HEPES, 25 mM glucose, 2 mM thiourea, 5 mM Na-ascorbate, 3 mM Na-pyruvate, 0.5 mM CaCl_2_·2H_2_O, and 10 mM MgSO_4_·7H_2_O. pH was titrated to 7.3–7.4 with 7 mL ± 0.2 mL of 37% hydrochloric acid, with osmolarity ranging from 300 to 305 mOsm/kg. The brain was quickly removed from the skull and placed in ice-cold NMDG-HEPES buffer. Acute brain slices containing the perirhinal cortex and hippocampus were prepared at a thickness of 275 μm using a vibratome (Leica VT1200 S, Wetzlar, Germany). Slices were incubated for 25 min at 35 °C and then transferred to a custom-made holding chamber at room temperature.

For experiments utilizing precision-cut lung slices (PCLS), lungs were harvested following euthanasia. The vasculature was first perfused through the left ventricle with PBS to clear erythrocytes, followed by intratracheal administration of 2% low-gelling temperature agarose and solidification at 4 °C for 1 h, as previously described.^40^ Next, 300 μm PCLS were generated using a vibratome [7000SMZ-2 Vibrotome (Campden Instruments, Ltd.)] into DMEM/F12 and 1% penicillin/streptomycin. Tissue punches (4 mm in diameter) containing visible airways were obtained from a PCL using a biopsy punch. After the O-PTIR and electrophysiology recordings, brain slices were fixed with 4% PFA at 4 °C overnight.

### Electrophysiology

Ex vivo whole-cell patch-clamp recordings were carried out on mouse brain slices. Neuronal cells were visualized using a fixed-stage Olympus Microscope (BX51WI) coupled with an IR-CCD camera and a 40× water immersion objective and recorded with continuous perfusion of artificial cerebrospinal fluid (aCSF, 1 mL/min) containing 124 mM NaCl, 2.5 mM KCl, 1.25 mM NaH_2_PO_4_, 24 mM NaHCO_3_, 12.5 mM glucose, 5 mM HEPES, 2 mM CaCl_2_·2H_2_O, and 2 mM MgSO_4_·7H_2_O; pH 7.4; 305 mOsm/kg. Borosilicate glass electrodes (4–6 MΩ resistance) were filled with intracellular solution containing 130 mM (K) gluconate, 10 mM KCl, 0.2 mM EGTA, 10 mM HEPES, 4 mM (Mg) ATP, 0.5 mM (Na) GTP, and 10 mM (Na) Phosphocreatine (pH 7.25, 296 mOsm). Recordings were obtained by using a Multiclamp 700B amplifier and pClamp 10.4 data acquisition software (Axon Instruments, Molecular Devices, USA). Original traces were obtained offline using Clampfit 10.6 (Axon Instruments, Molecular Devices, USA).

### *P. waltl* Imaging

The embryonic wild-type *P. waltl* [stage 34, 11 days post fertilization (dpf)] used in this study were from the colony at Lund University. Swedish regulations (Jordbruksverkets föreskrift L150, §5) state that working with vertebrate embryos before their ability to feed independently develops does not require Institutional Animal Care and Use Committee oversight. The stage selected is before the independent feeding stage. Embryos were reared in conditioned tap water at 20–22 °C (55 g Tetra marine salt, 15 g of Ektozon salt, and 2.5 mL of Prime water dechlorinator per 100 L of water). For imaging, embryos were removed from their protective jelly using fine-tip forceps at 10 dpf. At 11 dpf, corresponding to stage 34,^41^ embryos were narcotized in 0.025% of tricaine in conditioned tap water (pH 7.0). Once completely narcotized, embryos were then moved via a transfer pipet to a microscope slide for imaging. After imaging, embryos were then transferred back to the container with conditioned tap water (pH 7.0); all embryos were allowed to develop into larvae to assess for morphological damage.

### Spectroscopy

O-PTIR imaging was performed at Lund (integrated Vibration Spectroscopy–Microcosm Laboratory for Molecular-Scale Biogeochemical Research, Lund University). The IR source was a pulsed, tunable four-stage QCL device, scanning from 1800 to 800 cm^–1^ at a repetition rate of 100 kHz. The photothermal effect was detected through the modulation of the green laser (CW 532 nm) intensity induced by the pulsed IR laser. Further details about the fundamentals of the technique and the instrument itself can be found in the refs (51) and (52). To avoid the photodamage described for O-PTIR^24^ and use feasible settings for spectra collection, IR power was set to 22%, the IR pulse rate was set to 100 kHz, and Mirage DC was 1.005 V; green light probe power was 6% when the silicon photodiode detector (standard) had a gain of 10× or 0.6% for the avalanche photodiode detector (APD). These laser powers were selected because they provided the best signal-to-noise ratio and did not damage hydrated tissues. The hyperspectral map size for the fixed cells has a step size of 500 nm, which is the maximum resolution of the instrument. All of the O-PTIR spectra were collected in 3 scans. For the probe laser power set to 0.6%, the power on the sample was measured as ∼170 μm for the APD detector, and for the silicon photodiode detector, the power on the sample was measured as ∼4 mW. The power on the sample ranged from 0.06 to 0.77 mW from 1800 to 1200 cm^–1^ for the IR laser.

The spectra collection parameters were a spectral range of 1790–1000 cm^–1^ and a reflection mode at 2 cm^–1^ spectral resolution. Spectra were averaged for 5–8 scans. Background spectra were collected on a built-in reference sample. O-PTIR spectra were normalized to the maximum at 1656 cm^–1^; second-order derivation of the spectra was used to increase the number of discriminative features; the Savitsky–Golay algorithm with a 5-point filter with the third polynomial order was employed in this process. O-PTIR absorption images of living brain tissue were obtained with a linear scan speed of 100 μm/s with total acquisition times in the range of 2–3 min for 45 μm × 37 μm with the step of 250 nm. O-PTIR absorption images of embryos were obtained with a linear scan speed of 100 μm/s with total acquisition times in the range of 5 min for 100 μm × 50 μm with the step of 150 nm. The relative fraction of β-sheet structures was visualized by calculating the map intensity ratio between 1630 cm^–1^, a peak corresponding to β-sheet structures,^1,31,36^ and 1656 cm^–1^, the maximum of Amide I.^1^ The increase in intensity in the resultant ratio map was considered a sign of the higher concentration of amyloid fibrils. The intensity at 1740 cm^–1^ was considered a sign of the higher concentration of esters.^43^

### Tissue Biopsies and Histological Assessment

After brain extraction, the animal’s chest and abdominal cavities were opened, and all organs (heart, lung, liver, kidney, and salivary gland) were removed and placed on a petri dish on ice. Tissue biopsies were obtained using a 2 mm biopsy punch (WellTech, Denmark) and were approximately 1.5 mm in height. Biopsies were placed in 96-well plates containing complete cell culture media [DMEM/F12 (Gibco, Sweden) cell culture media supplemented with 0.1% FBS (Gibco, Sweden), 1% penicillin/streptomycin (Gibco, USA), and 1% AmpB (Amphotericin B, Gibco, UK)].

After spectroscopy and the metabolic viability assay (described below), the tissue was fixed overnight at 4 °C in 10% neutral buffer formalin solution (Sigma-Aldrich, Sweden) and processed as previously described.^53^ Specifically, formalin-fixed tissue was passed through graded ethanol (Solveco, Sweden) and isopropanol (Univar Solutions, Sweden) solutions prior to paraffin embedding (Histolab, Sweden) in an automated spin tissue processor (Myr, Spain) using processing/embedding cassettes (Histolab, Sweden).^53^ Paraffin-embedded tissue biopsies were cut into 4 μm tissue sections using a microtome (Leitz, Germany) and mounted on Superfrost adhesive microscope slides (Epredia, USA). Before chemical deparaffinization, slides were placed in a horizontal position overnight in a 65 °C oven, followed by deparaffinization using Histo-Clear (National Diagnostics, USA). After deparaffinization, tissue slides were stained with hematoxylin and eosin (Merck Millipore, Germany), dehydrated, mounted with Pertex mounting media (Histolab, Sweden), and imaged using a virtual microscopy slide scanning system (VS120-S6–096, Olympus, Tokyo, Japan).

### Metabolic Assessment of Tissue Slices

Tissue punches were randomly divided into two groups: nonexposed tissue (control) and tissue that had been IR-irradiated and cultured in complete media. Metabolic activity (i.e., tissue viability) was assessed using the water-soluble and cell-permeable tetrazolium salt reagent (WST-1), which is reduced on the membrane of mitochondria in living cells.^54^ Following intracellular cleavage of WST-1 by cellular mitochondrial dehydrogenases into formazan, the cell-permeable formazan is released into the cell culture media and can subsequently be measured by removing the liquid and reading it in a standard UV–vis plate reader. Absorbance can generally be viewed as proportional to the metabolic activity of living cells. Therefore, lower amounts of formazan indicate a reduced metabolic activity. Tissue slices or biopsy pieces were incubated with 90 μL of complete medium and 10 μL of WST-1 (Roche, Sigma-Aldrich, USA) for 1 h at 37 °C in a humidified incubator with 5% CO_2_. Supernatant optical density was measured at 440 and 650 nm on an Epoch plate reader (BopTeck, USA). 440 nm was used as a representative for the specific conversion of WST-1 by metabolically active cells, and 650 nm wavelength was used as a reference wavelength for nonspecific absorbance. Complete medium without tissue served as a negative control.

### Tissue Labeling

After O-PTIR imaging, the presence of amyloid plaques in fresh APP/PS1 brain tissue was confirmed by staining with Amytracker^R^ 520 (EbbaBiotech, Solna, Sweden). According to the manufacturer’s protocol, Amytracker^R^ 520 was diluted from 1 to 1000 in the presence of DAPI diluted from 1 to 10000, and living tissue slices were incubated for 30 min at room temperature in a 24-well plate.

Tissue labeling with 82E1 (IBL-America, Minneapolis, USA), Map2 (M13, Invitrogen/Thermo Fisher Scientific, Sweden) Monoclonal Antibody, and AnnexinV Alexa Fluor568 conjugate (A13202, Invitrogen/Thermo Fisher Scientific, Sweden) was performed according to the manufacturer’s protocols, after tissue fixation and permeabilization with 0.5% Triton and 0.1% SDS during 24 h. DAPI was added at the last step of tissue labeling using dilution from 1 to 10000 for 10 min of incubation at room temperature; after that, the tissue was mounted on glass slides and imaged.

### Live Imaging of Cellular Damage in Precision-Cut Lung Slices Following O-PTIR Imaging

Immediately after vibratome slicing and prior to O-PTIR imaging, individual 4 mm PCLS were cultured in 100 μL of complete medium in a culture-insert 4-well plate (Ibidi, Germany). PCLS that were not exposed to O-PTIR imaging were treated identically, including transport to the imaging facility, and served as controls. PCLS slices were placed on histological glass for O-PTIR imaging, and high spectral maps at 1656 cm^–1^ were acquired from selected identifiable airways. After O-PTIR imaging, PCLS were placed in a 37 °C cell culture incubator overnight, as described above. After 24 h, PCLS were stained using the NucView488 and RedDot2 Apoptosis & Necrosis kit (Biotium, USA) according to the manufacturer’s protocol to visualize cellular damage. Identical settings were used for all images, including *z*-stack acquisition (∼128 μm total thickness and *z*-step of 2.4 μm), using a STELLARIS confocal microscope (Leica, Germany), and using FITC and Cy5 laser settings of 15.9% and 6.7% excitation intensity and 107.6 and 29.5 gain, respectively. Airways imaged by O-PTIR were identified manually by correlation of bright-field images and STELLARIS microscopes. Maximum intensity projections were rendered using the maximum intensity function in Leica LAS X software.

Ciliary beating was assessed from the same airways pre- (“0 h”) and post-O-PTIR imaging (24 and 48 h) with a Nikon TS-2r microscope equipped with a DFK NME33UX264 camera. Airways were imaged using a 20× objective at 30 Hz and an image size of 2488 × 2048 pixels.

### LDH Release (Quantitative Cell Membrane Damage)

The release of lactate dehydrogenase (LDH) was assessed according to the manufacturer’s protocol using the Cytotoxicity Detection Kit (Roche, Germany). Briefly, 10 μL of supernatant culture media from 4 mm PCLS was collected 30 min after slicing and prior to O-PTIR imaging (“0 h”) and after 24 and 48 h and then stored at −20 °C until analysis. For analysis, 2 μL of collected supernatant or fresh cell culture media (i.e., background control) was gently mixed (to avoid bubbles) with 98 μL of LDH reaction mixture. The mixture was incubated for 1 h at room temperature, shielded from direct light. Subsequently, the optical density was measured at 492 nm with a reference wavelength of 650 nm using a multiwell plate reader (Epoch, BioTek, USA).

### Cytation 5 Tissue Imaging

Tissue imaging was acquired using the manual mode in a Cytation 5 multimode reader (Agilent Technologies, USA). Montage images were collected at 4× magnification. Texas red and DAPI imaging filter cubes were used with the following acquisition settings: DAPI: LED intensity: 3, integration time: 13 ms, camera gain: 24. Texas red: LED intensity: 3, integration time: 97 ms, camera gain: 18.

### Confocal Microscopy

A laser scanning Leica TCS SP8 confocal microscope (Leica Microsystems) with Leica Application Suite X software (version 3.4.7.23225) was used to obtain high-resolution images of amyloid plaques. 5× air and 40× oil objectives in combination with a 552 laser and a *z*-step size of 1.0 μm were used to acquire the fluorescent images.

## Data Availability

All data and materials used in the analysis are available in some form to any researcher for purposes of reproducing or extending the analysis.

## Supporting Information Available

Cilia movement videos (AVI)
Representative embossed contrast videos of cilia beating in the airways of PCLS 48h after O-PTIR irradiation (AVI)
Bright-field images, metabolic activity, and H&E staining of tissue biopsies with and without O-PTIR imaging, including corresponding O-PTIR acquired spectra (brain and liver); overview of brain slice sample support; electrophysiology recordings of brain tissue slices after O-PTIR imaging; hyperspectral map with corresponding spectra and histochemical staining of amyloid plaques in living brain slices; histochemical staining of cellular damage over time (brain and lungs) and LDH release; and bright-field images of an axolotl’s skin and spectra of lipid droplets (PDF)

## Supplementary Material

ja3c08854_si_001.avi

ja3c08854_si_002.avi
