# Supplementary material for: Label-Free High-Resolution Photothermal Optical Infrared Spectroscopy for Spatiotemporal Chemical Analysis in Fresh, Hydrated Living Tissues and Embryos
Source: J Am Chem Soc. 2023 Nov 2;145(45):24796–808. doi: 10.1021/jacs.3c08854 (PMC10655180; doi:10.1021/jacs.3c08854)
Supplement: Supplementary file 3 — ja3c08854_si_003.pdf [file ja3c08854_si_003.pdf]

# Label-Free High-Resolution Photothermal Optical Infrared Spectroscopy for Spatiotemporal Chemical Analysis in Fresh, Hydrated Living Tissues and Embryos

Nika Gvazava,[†§⊥#] Sabine C. Konings,[†‡§] Efrain Cepeda-Prado,[†‡⊥] Valeriia Skoryk,[†‡§] Chimezie H.Umeano,[∥⊥#] Jiao Dong,[§⊥#] Iran A. N.Silva,[§⊥#] Daniella Rylander Ottosson,[†‡⊥] Nicholas D. Leigh,[∥⊥#] Darcy Elizabeth Wagner,[†§⊥#] Oxana Klementieva[*†‡§]

† Department of Experimental Medical Science, Lund University, 22180 Lund, Sweden

‡ MultiPark, Lund University, 22180 Lund, Sweden

§ NanoLund, Lund University, 22180 Lund, Sweden

∥ Department of Laboratory Medicine, Molecular, Medicine and Gene Therapy, 22184 Lund, Sweden

⊥ Lund Stem Cell Center, Lund University, 22100 Lund, Sweden

# Wallenberg Centre for Molecular Medicine, Lund University, 22184 Lund, Sweden

*Corresponding author. Email: Oxana.klementieva@med.lu.se

Keywords: Photothermal, optical, infrared, living tissue, nanospectroscopy

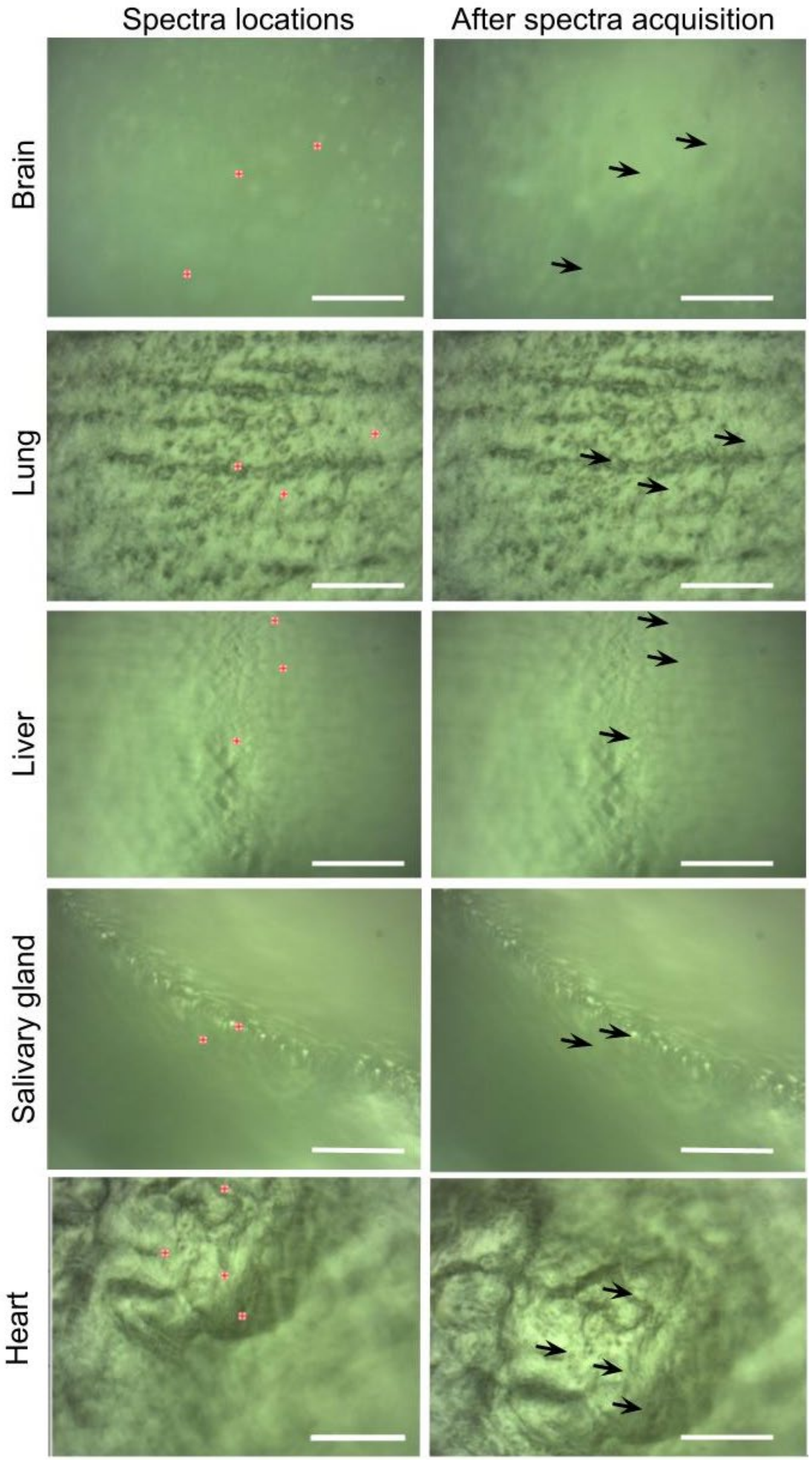


**Figure S1. Bright-field view of tissues before and after spectroscopy**. Red crosses indicate spectra acquisition locations; black arrows show the spots after spectra acquisition. Scale bar is 40 μm in all subpanels.

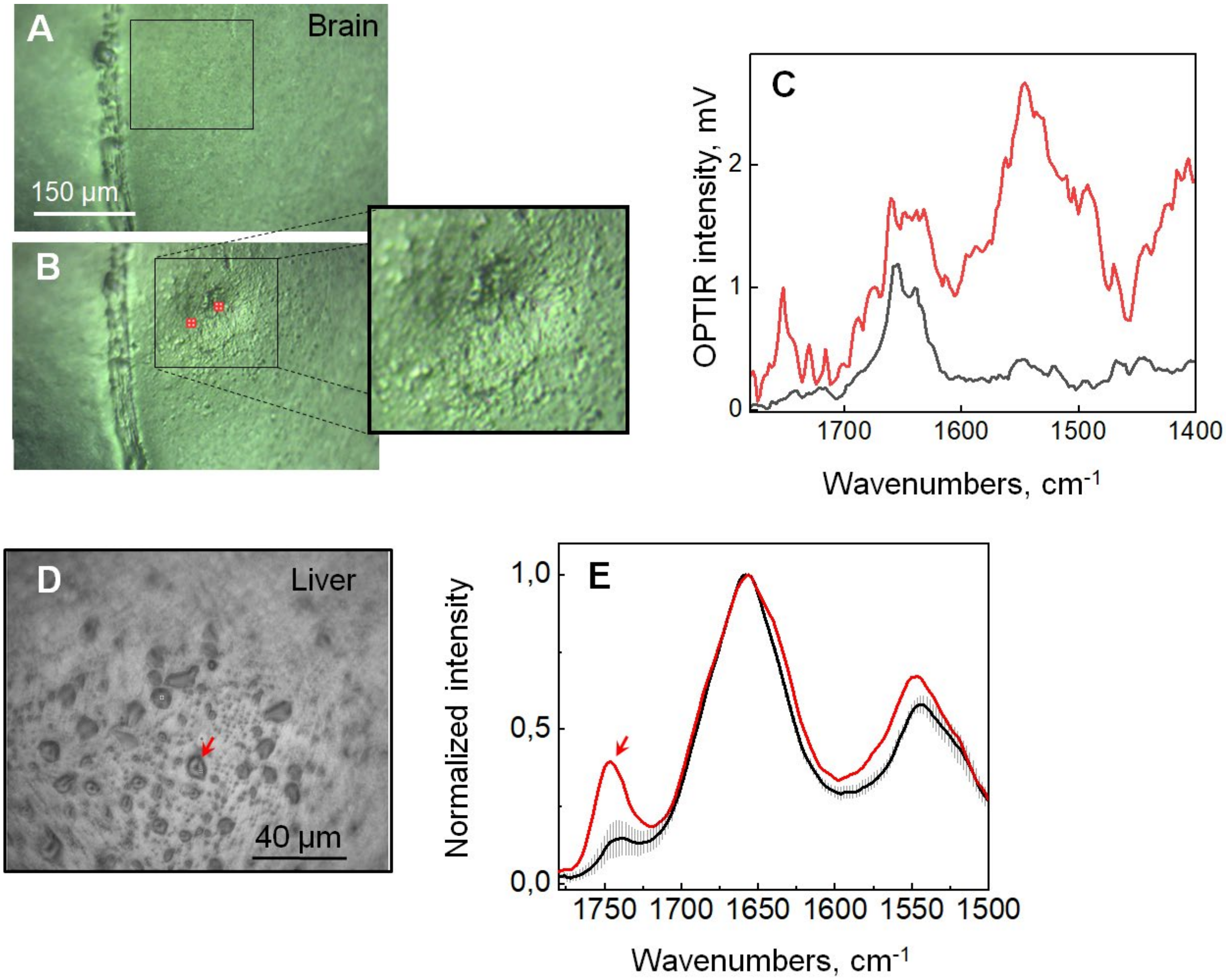


**Figure S2. O-PTIR spectroscopy of brain and liver tissue.** (**A**) Bright-field view image of brain tissue before spectroscopy. Red dots indicate spectra locations. (**B**) Bright-field view image of brain tissue after spectra acquisition. Black arrow shows the tissue damage. Insert corresponds to a digitally zoomed image of damaged area. (**C**) Example of O-PTIR spectra with altered Amide I/II ratio due to tissue damage by high QCL intensity. Black and red spectra demonstrate altered Amide II intensity (1550 $cm^{-1}$). (**D**) Bright-field view image of liver tissue. Red arrow shows a location for O-PTIR spectra. (**E**) Normalized O-PTIR spectrum, arrow shows the peak centered at 1740 cm-1 as compared to averaged spectra shown in Fig.1E.

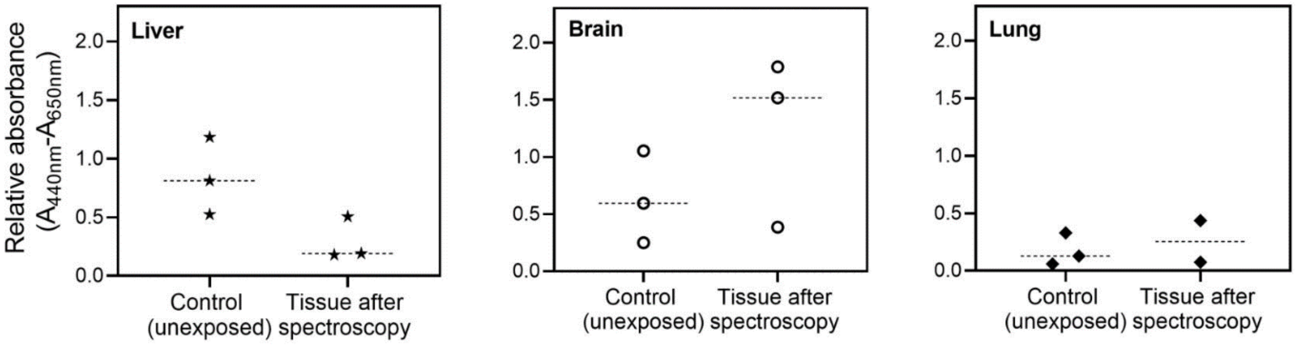


**Figure S3. Water-soluble tetrazolium-1 tissue viability assay.** Tissue viability after O-PTIR measurement, as assessed using WST1. Identically prepared and time-matched tissues that were not exposed to spectroscopic measurements were used as controls. During O-PTIR measurements, control biopsies were in the organ-specific analysis buffer. Tissue biopsies subjected to O-PTIR spectroscopy were placed back in 24-well with DMEM/F12 media. All samples were then incubated for 1 hour in the presence of a water-soluble tetrazolium salt reagent. Absorbance was then read at 440 nm ($A_{440nm}$), with absorbance at 650 nm serving as a non-specific reference wavelength ($A_{650nm}$). Each data point represents an individual biopsy with the dashed line indicating the mean (n=3 for all conditions, except lung tissue, where one sample was lost due to a technical error after spectroscopy) and is presented as relative absorbance ($A_{440nm}$-$A_{650nm}$). No statistical difference was found (Mann-Whitney U test). All samples were above background (i.e. absorbance determined for each organ-specific analysis buffer with added WST1, incubated at the same concentration as in the assay).

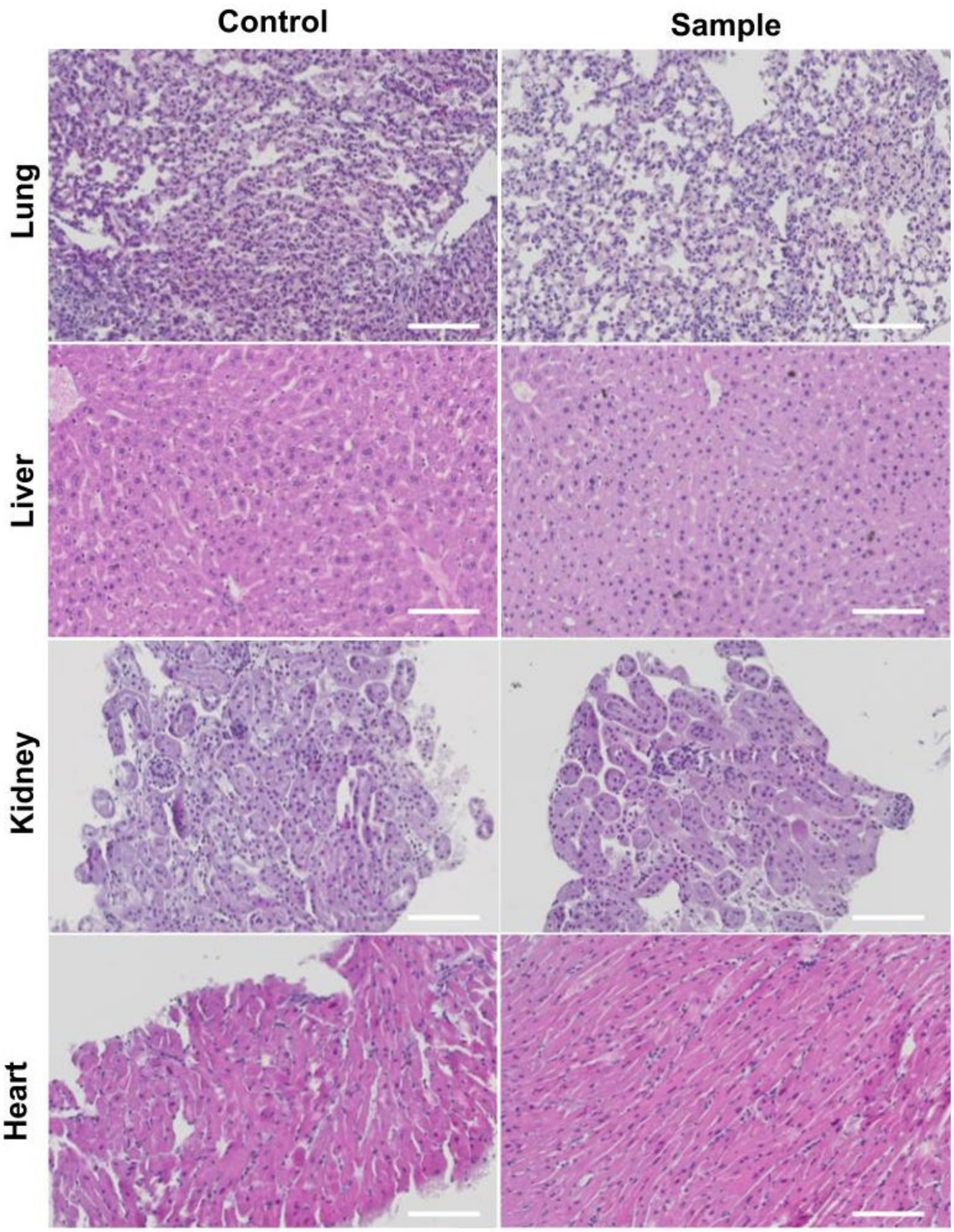


**Figure S4. Post hoc histological analysis.** After spectroscopy, tissue biopsies were fixed in neutral buffered formalin, dehydrated, embedded in paraffin, and sectioned for hematoxylin-eosin tissue staining. Control biopsies are samples that were not analyzed by O-PTIR (Control) and tissue biopsies after O-PTIR (sample). Scale bar is 100 μm in all subpanels.

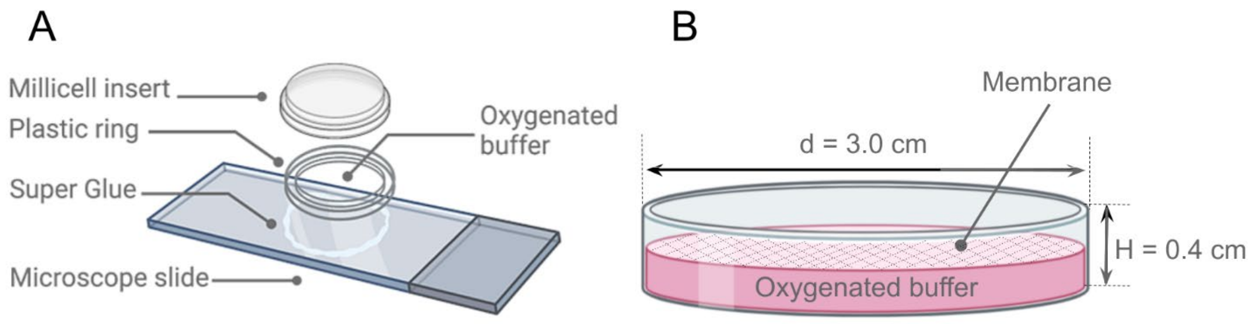


**Figure S5. Sample support overview**. The sample chamber was designed to fit into the MIRAGE microscope for the measurements in refractive mode, adapting the chamber dimensions so that the MIRAGE objective can move to focus on the z-axis and also in the x and y directions. (**A**) The home-made recording chamber was constructed using two stackable Millicell Cell Culture Inserts. The lower compartment was made using a Millicell insert without a membrane (30mm plastic ring) and attached to a microscope slide with Super Glue (Loctite, 2062278, USA). Next, the lower chamber was filled with 2.5 ml of pre-oxygenated (95% oxygen, 5% carbon dioxide gas) artificial cerebrospinal fluid at room temperature. The upper part of the chamber corresponds to another Millicell insert stacked on top of the lower plastic ring. This chamber was removable and contained a hydrophilic polytetrafluoroethylene (PTFE) membrane with 0.4 µm pore size (Millipore, PICM0RG50, Ireland) to allow for media exchange between the two chambers. (**B**) The tissue sample rests on the insert's membrane, creating a buffer-tissue interface that keeps the sample hydrated and alive for approximately 20 minutes, enough to complete the O-PTIR imaging.

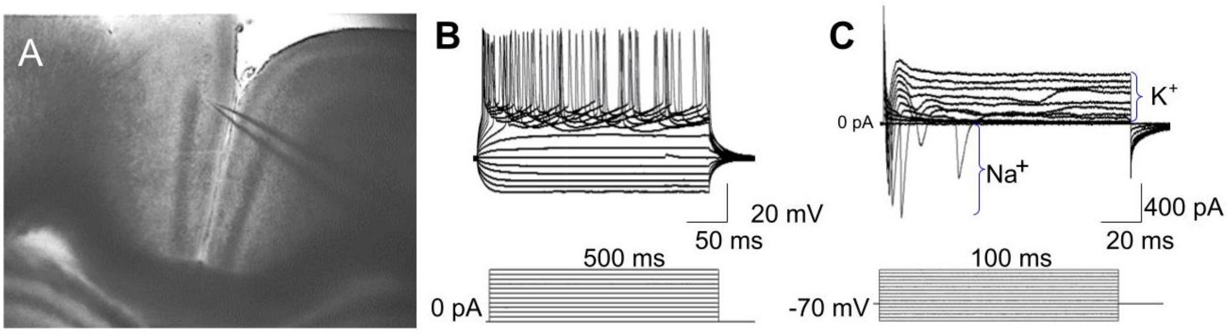


**Figure S6. Electrophysiology after O-PTIR.** (**A**) The tissue overview was visualized with differential interface contrast imaging. (**B**) Induction of action potentials (firing pattern) to increasing current injection (from –100 pA to 200 pA with a delta of 20 pA for 500 ms, see below), recorded in current clamp mode at a holding potential of –70 mV. (**C**) Traces show voltage-dependent sodium (Na + ) and potassium (K + ) currents evoked by increasing depolarizing voltage steps from −70 mV to 40 mV with a delta of 10 mV for 100 ms in voltage-clamp mode.

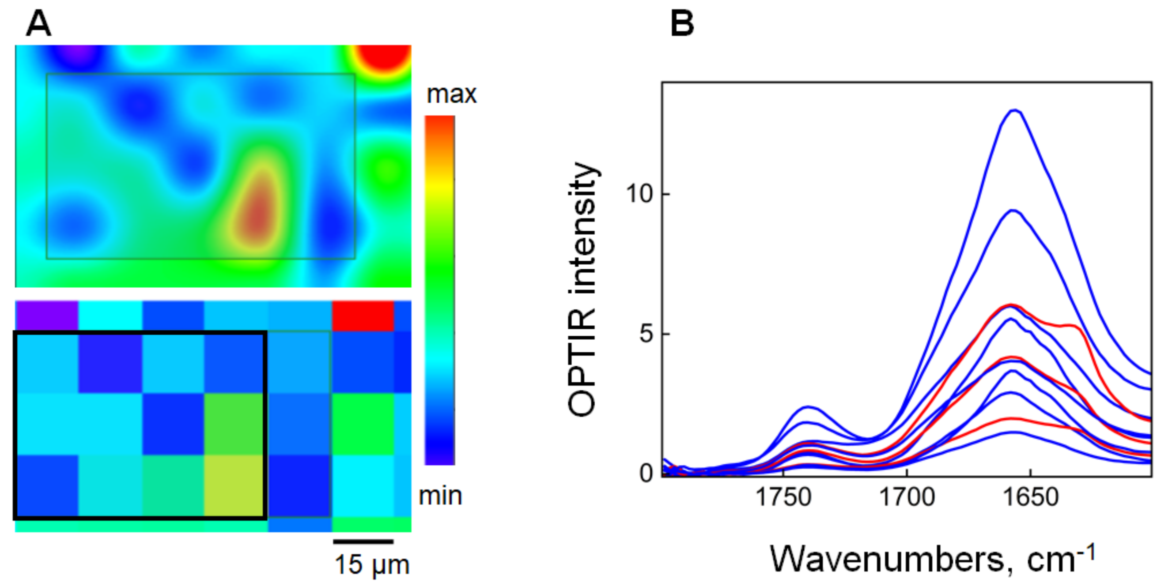


**Figure S7. Hyperspectral imaging of amyloid plaques in a mouse brain tissue model of AD. (A)** The upper panel interpolated hyperspectral map of 1630 cm$^{-1}$ is normalized to 1656 cm$^{-1}$, and the lower panel is a corresponding pixel map taken at the 15-micron step. (**B**) Corresponding raw O-PTIR spectra.

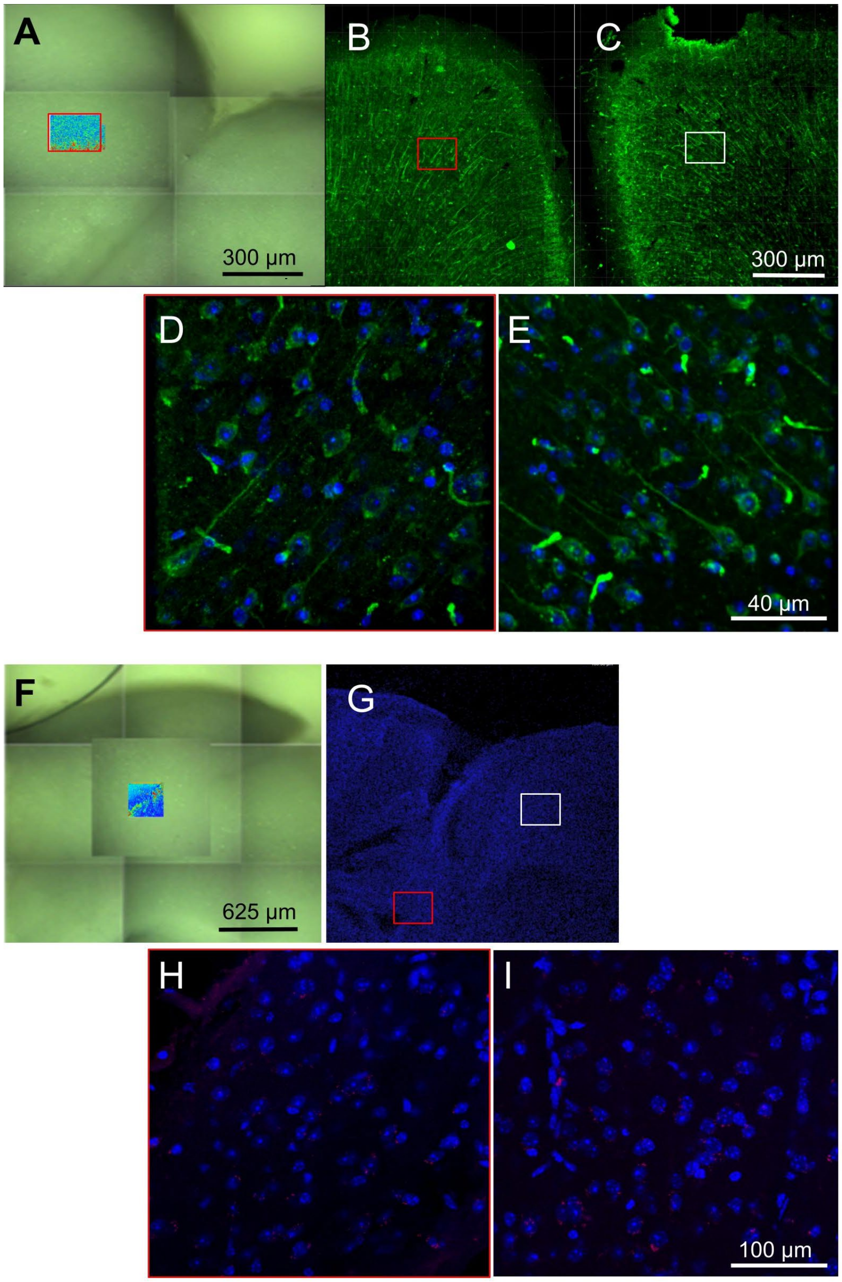


**Figure S8. Tracking of cellular damage in brain slices following O-PTIR imaging.** (**A**) Bright-field image displaying the location of a single energy map acquired at 1656 cm$^{-1}$ in RSC (the insert). (**B, C**) Confocal images of MAP2-positive neurons (green) in the RSC irradiated area (B) and the unexposed hemisphere (C). A red rectangle indicates the approximate location of O-PTIR measurements. (**D, E**) High-resolution confocal images showing MAP2-positive neurons retaining their morphology after the spectroscopic measurements compared to the unexposed tissue. (**F**) Another bright field image shows the location of a single energy map acquired at 1656 cm$^{-1}$ in RSC (the insert). (**G**) Overview DAPI image of the same RSC after O-PTIR. The approximate location of O-PTIR measurements is indicated by the red rectangle. (**H, I**) High-resolution images of AnnexinV (red dots) in (H) tissue after spectroscopy and (I) unexposed tissue.

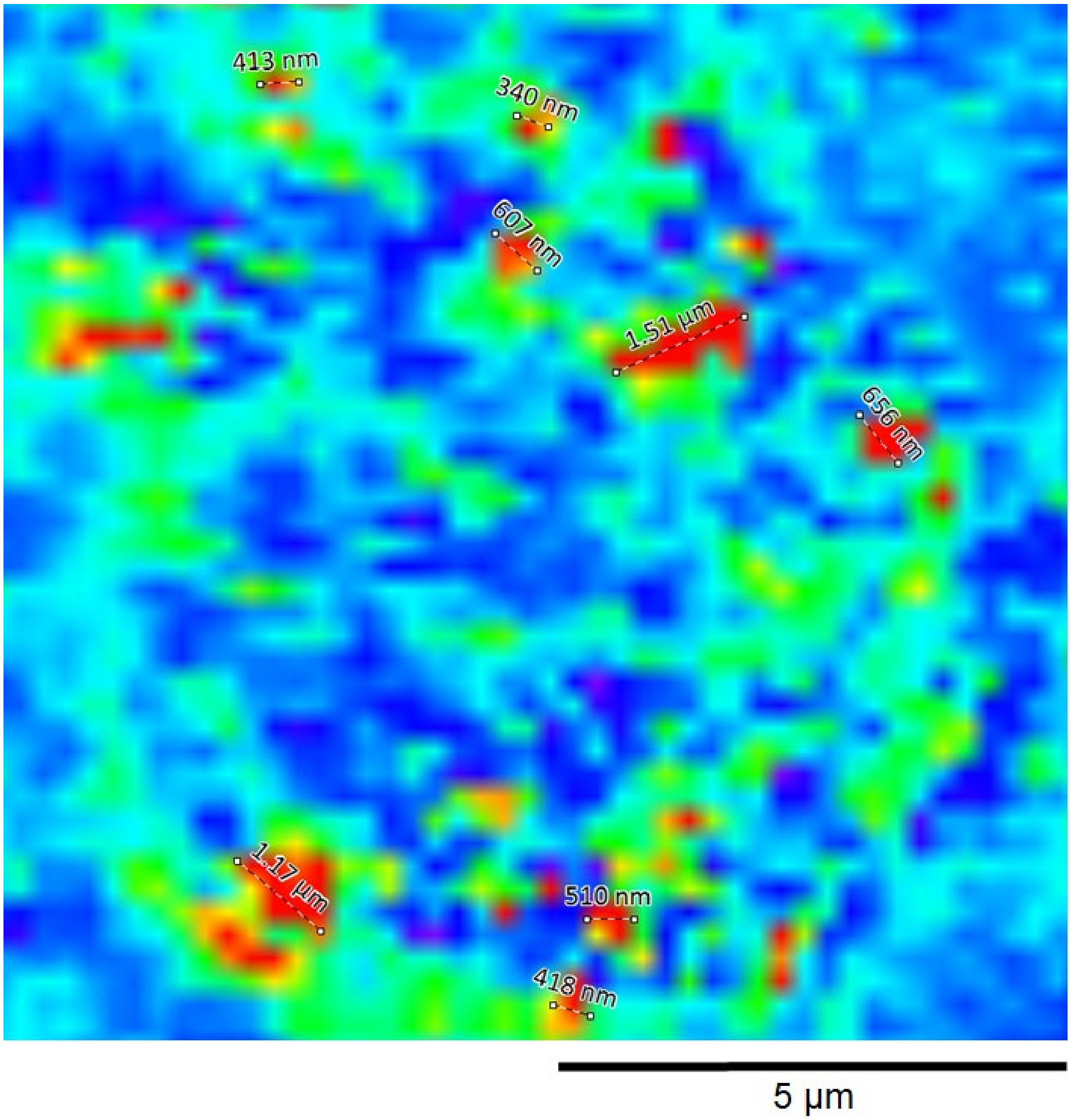


**Figure S9.** Distribution of β-sheet structures calculated as the ratio of O-PTIR maps measured at 1630 to 1656 $cm^{-1}$ in living mouse brain tissue as detected by O-PTIR. The size of aggregates is measured in the O-PTIR software.

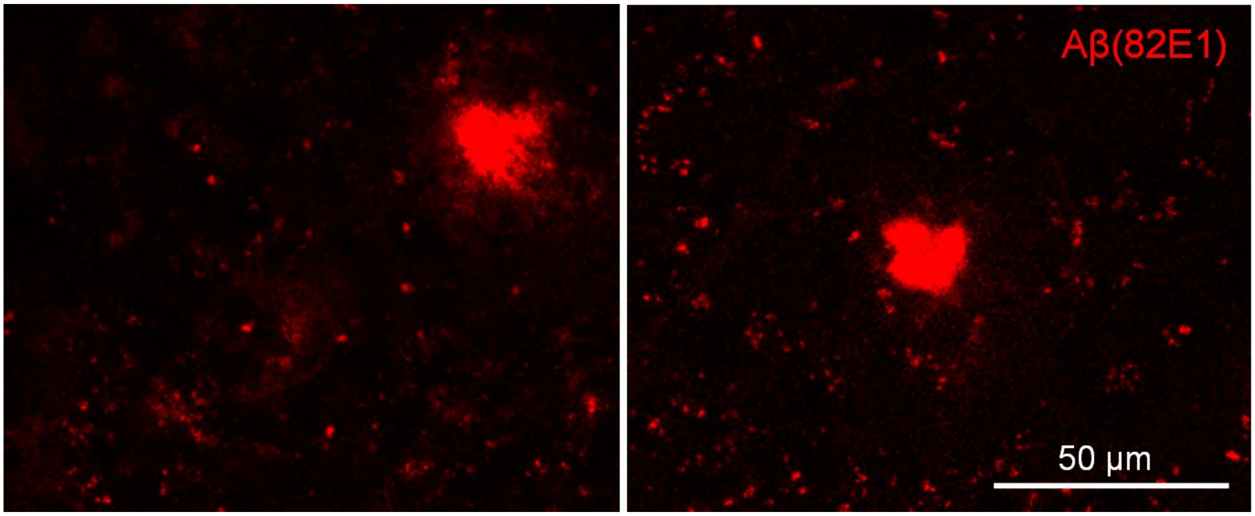


**Figure S10.** Confocal image of amyloid plaques in brain tissue showing amyloid proteins immunolabeled with Aβ specific antibody (82E1, red). Left panel: tissue fixed immediately after cutting brain slices. Right panel: tissue fixed after O-PTIR.

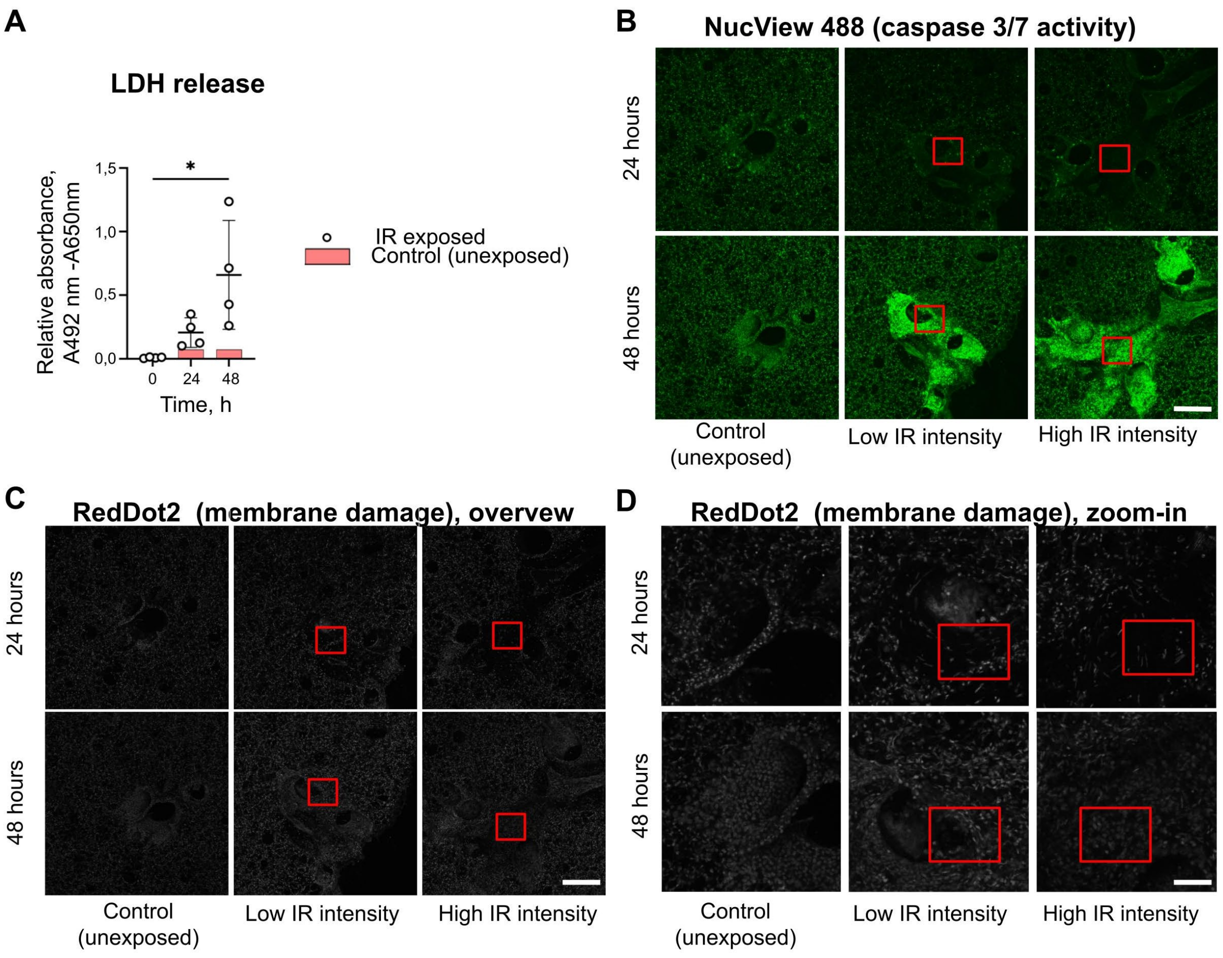


**Figure S11. Live tracking of cellular damage in precision-cut lung slices (PCLS) following O-PTIR imaging.** (**A**) LDH assay. (**B-D**) Maximum projection intensity from z-stacks acquired with confocal microscopy and stained live (**B**) NucView 488 (fluorescent DNA dye coupled to a peptide sequence recognized by active caspase 3/7) and (**C-D)** RedDot2 (far-red dye for membrane compromised or dead cells). Red insets represent the areas imaged with O-PTIR at different laser intensities and tracked over time for 24 and 48 hours, showing subjective increases in staining at 48 hours in samples subjected to O-PTIR imaging. Control samples represent similarly cultured and transported samples not exposed to O-PTIR. Scale bar is 100 μm in all subpanels.

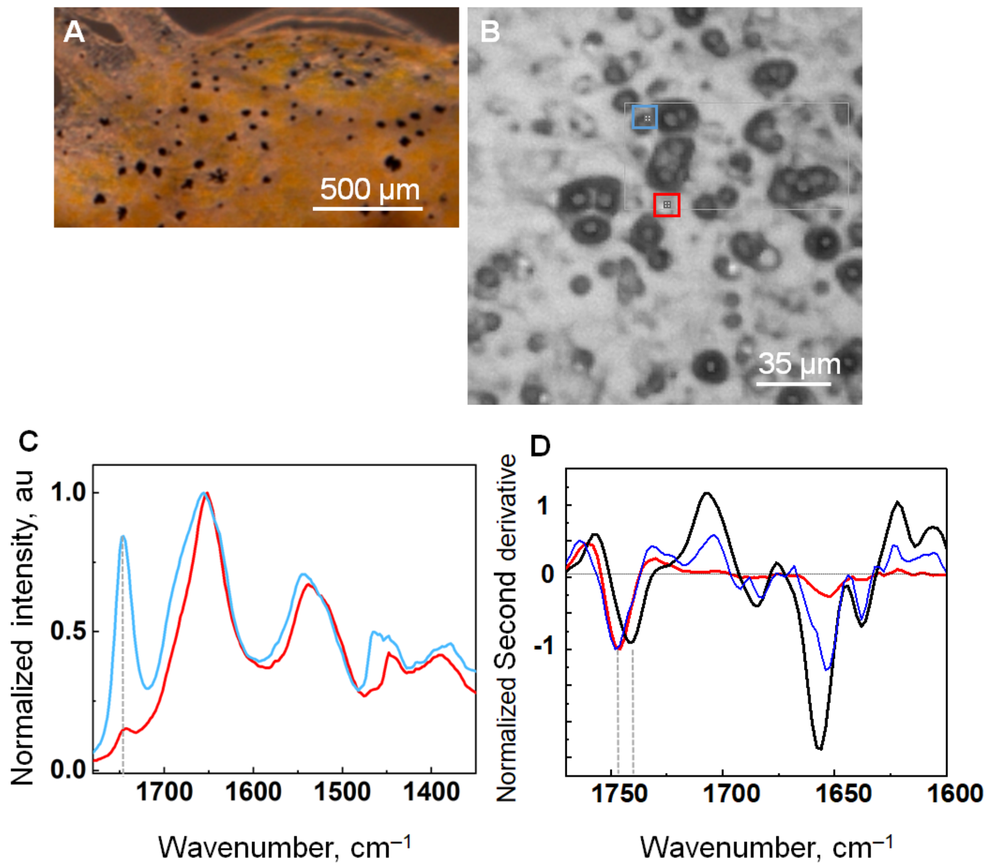


**Figure S12. O-PTIR on *Pleurodeles waltl.*** (**A**) Sample overview of the *Pleurodeles waltl* embryonic skin. (**B**) Spectra locations were acquired from the droplets on the *Pleurodeles waltl* embryonic skin. (**C**) Normalized O-PTIR spectra. The dashed line indicated the band located at 1750 $cm^{-1}$ corresponding to ester −C═O, −C═OOH groups. (D) Normalized to 1740 max and averaged second derivatives of spectra from mouse skin tissue, red spectrum was acquired from dried *Pleurodeles waltl* skin; blue spectra from living *Pleurodeles waltl*. Dashed lines indicate peak positions.

**Supplementary Video 1-2.** Representative embossed contrast videos of cilia beating in the airways of PCLS 48h after OPTIR irradiation. Videos were captured in living PCLS using a Nikon TS-2r microscope and 20x objective with a framerate of 30 Hz. Supplementary video 1 represents an airway irradiated with high (48%) IR intensity. Supplementary video 2 represents airway irradiated with lower (22%) IR power. White arrow points to beating cilia.
